# Utilizing *TP53* hotspot mutations as effective predictors of gemcitabine treatment outcome in non-small-cell lung cancer

**DOI:** 10.1038/s41420-025-02300-7

**Published:** 2025-01-27

**Authors:** Yen-Han Tseng, Trieu Thi My Tran, Jinghua Tsai Chang, Yu-Tang Huang, Anh Thuc Nguyen, Ian Yi‑Feng Chang, Yi-Tung Chen, Hao-Wen Hsieh, Yue-Li Juang, Peter Mu-Hsin Chang, Tzu-Yi Huang, Ying-Chih Chang, Yuh-Min Chen, Hsuan Liu, Chi-Ying F. Huang

**Affiliations:** 1 Department of Chest Medicine, Taipei Veterans General Hospital, Taipei, Taiwan; 2 Program in Molecular Medicine, College of Life Sciences, National Yang Ming Chiao Tung University, Taipei, Taiwan; 3 School of Medicine, College of Medicine, National Yang Ming Chiao Tung University, Taipei, Taiwan; 4 Institute of Biopharmaceutical Sciences, College of Pharmaceutical Sciences, National Yang Ming Chiao Tung University, Taipei, Taiwan; 5 Institute of Biomedical Sciences, Academia Sinica, Taipei, Taiwan; 6 Institute of Medicine, Chung Shan Medical University, Taichung City, Taiwan; 7 Biomedical Industry Ph.D. Program, College of Life Sciences, National Yang Ming Chiao Tung University, Taipei, 112304 Taiwan; 8 Program in Molecular Medicine, Taiwan International Graduate Program in Molecular Medicine, National Yang Ming Chiao Tung University and Academia Sinica, Taipei, Taiwan; 9 Molecular Medicine Research Center, Chang Gung University, Taoyuan, Taiwan; 10 Department of Neurosurgery, Lin-Kou Medical Center, Chang Gung Memorial Hospital, Taoyuan, Taiwan; 11 Institute of Clinical Medicine, School of Medicine, National Yang Ming Chiao Tung University, Taipei, Taiwan; 12 Institute of Biomedical Sciences, Mackay Medical College, New Taipei City, Taiwan; 13 Department of Oncology, Taipei Veterans General Hospital, Taipei, Taiwan; 14 Genomics Research Center, Academia Sinica, Taipei, 115 Taiwan; 15 Department of Chemical Engineering, Stanford University, Stanford, CA USA; 16 Department of Cell and Molecular Biology, College of Medicine, Chang Gung University, Taoyuan, Taiwan; 17 Graduate Institute of Biomedical Sciences, College of Medicine, Chang Gung University, Taoyuan, Taiwan; 18 Division of Hematology-Oncology, Department of Internal Medicine, Chang Gung Memorial Hospital at Linkou, Taoyuan, Taiwan; 19 Department of Biochemistry, School of Medicine, Kaohsiung Medical University, Kaohsiung, Taiwan; 20 Chong Hin Loon Memorial Cancer and Biotherapy Research Center, National Yang Ming Chiao Tung University, Taipei, Taiwan

**Keywords:** Cell death, Cancer epigenetics, Non-small-cell lung cancer

## Abstract

*TP53* mutations are recognized to correlate with a worse prognosis in individuals with non-small cell lung cancer (NSCLC). There exists an immediate necessity to pinpoint selective treatment for patients carrying *TP53* mutations. Potential drugs were identified by comparing drug sensitivity differences, represented by the half-maximal inhibitory concentration (IC50), between *TP53* mutant and wild-type NSCLC cell lines using database analysis. In addition, clinical data from NSCLC patients were collected to evaluate both their *TP53* status and their response to gemcitabine, thereby facilitating further validation. Subsequently, NSCLC cell lines with different *TP53* status (A549 and H1299) were subjected to gemcitabine treatment to investigate the association between *TP53* mutations and gemcitabine response. According to the dataset, NSCLC cell lines carrying *TP53* mutations displayed heightened sensitivity to gemcitabine. From a clinical standpoint, patients exhibiting *TP53* hotspot mutations demonstrated prolonged overall survival upon gemcitabine treatment. In vitro, overexpressing various hotspot *TP53* mutations significantly sensitized H1299 cells to gemcitabine. Moreover, the knockdown of *TP53* in A549 cells notably augmented sensitivity to gemcitabine treatment, as evidenced by cell viability and reproductive cell death assays. Conversely, the overexpression of wild-type *TP53* in H1299 cells led to an increased resistance against gemcitabine. Gemcitabine is a treatment option for patients with non-small cell lung cancer (NSCLC) who carry *TP53* hotspot mutations. This potential effectiveness might arise from its ability to disrupt DNA damage repair processes, leading to G2/M phase cell cycle arrest or an augmentation of mitotic abnormalities, eventually cause cell death. As a result, when planning treatment strategies for NSCLC patients possessing *TP53* hotspot mutations, gemcitabine should be considered to incorporate into the indication.

## Introduction

Lung cancer is the leading cause of cancer death worldwide [1]. Genetic alternations wield a pivotal influence over lung cancer treatment strategies. Targeted therapies, such as EGFR, ALK, and ROS1 inhibitors, have become the standard of care for actionable gene alterations. Nevertheless, the presence of other mutations, like *TP53*, might curtail the responsiveness to targeted interventions. Notably, *TP53* mutations have been detected in around 52% of non-small cell lung cancer (NSCLC) patients. Although certain early clinical trials are currently evaluating the efficacy of *TP53*-mutant inhibitors, their clinical applicability remains pending [2].

*TP53*, a tumor suppressor gene, plays a crucial role in forestalling malignant cell formation. Research has indicated that *TP53* mutations can manifest at varying phases of cancer initiation and progression [3]. In some instances, *TP53* mutations may be present in the early stages of cancer development and act as an initiating event. In other cases, these mutations may occur later in the process and contribute to the progression and metastasis [4]. A noteworthy association has been established between *TP53* mutations and unfavorable prognoses, as well as an elevated probability of treatment resistance [5, 6]. Consequently, comprehending the impact of *TP53* mutations on cancer development and progression has emerged as a pivotal focus within oncological research [7].

*TP53* hotspot mutations are specific alterations that occur at certain codon residues in the *TP53* gene, which encodes the p53 tumor suppressor protein. These hotspot mutations are observed in various human cancers [8]. The most frequently occurring *TP53* hotspot mutations are distributed in the DNA-binding domain of the p53 protein, which is essential for its tumor suppressor function. These mutations can disrupt the DNA-binding ability of the p53 protein, leading to the loss of its ability to regulate the cell cycle and prevent the formation of cancer cells. The most common *TP53* hotspot mutations include R175H, R248W, R273H, and R282W, which occur at residues 175, 248, 273, and 282, respectively [9]. These mutations have been extensively studied and have been shown to be associated with a wide range of cancer types. Understanding the specific effects of *TP53* hotspot mutations on the function of the p53 protein is crucial for developing targeted cancer therapies that can effectively treat cancers harboring these mutations. Therefore, ongoing research in this area is critical for advancing our understanding of cancer biology and for developing novel therapeutic strategies for cancer treatment.

Concurrently with the quest for *TP53* mutation inhibitors, an alternative avenue involves the identification of existing drugs capable of targeting these mutations, either directly or indirectly. In the CONKO-001 phase III clinical trial, sequencing data from patients indicated that *TP53* mutations could serve as predictive indicators for gemcitabine treatment efficacy in pancreatic cancer [10]. This discovery serves as a pivotal launching point for further substantiating the gemcitabine-*TP53* mutations relationship across diverse cancer types. Moreover, it provides insights into the underlying mechanics of this drug-mutation interplay.

Gemcitabine, a nucleoside analog of deoxycytidine (2,2-difluorodeoxycytidine, dFdC), inhibits cell proliferation during interphase via masked chain termination, thus interfering with DNA synthesis [11]. It is widely used as a chemotherapeutic agent in combination with other cytotoxic agents for the treatment of various types of cancer [11]. Gemcitabine monotherapy or in combination with cisplatin has achieved a response rate of 18–26% in advanced NSCLC patients [12]. Working as a prodrug, gemcitabine necessitates cellular uptake and subsequent phosphorylation into triphosphate difluorodeoxycytidine to integrate into the DNA chain during the S phase. Research has indicated correlations between altered SLC29A1, CDA, and RRM1 protein metabolism and patient responsiveness to gemcitabine treatment. The expression of hENT1, a cellular transporter facilitating gemcitabine entry, has emerged as a potential prognostic biomarker for gemcitabine-based therapies in pancreatic ductal adenocarcinoma patients [13, 14].

In this study, our objective encompassed unraveling gemcitabine’s potential as a viable therapeutic avenue for NSCLC patients bearing *TP53* hotspot mutations. We embarked on this study by analyzing drug sensitivity and genetic alterations within comprehensive databases. Subsequently, patients diagnosed with NSCLC and subjected to gemcitabine treatment were enlisted for validation. Finally, distinct NSCLC cell lines were employed to corroborate our findings functionally and delve into the underlying mechanisms.

## Results

### From the database analysis, gemcitabine emerged as a promising anticancer agent for NSCLC cell lines bearing *TP53* mutations

**Fig. 1:**
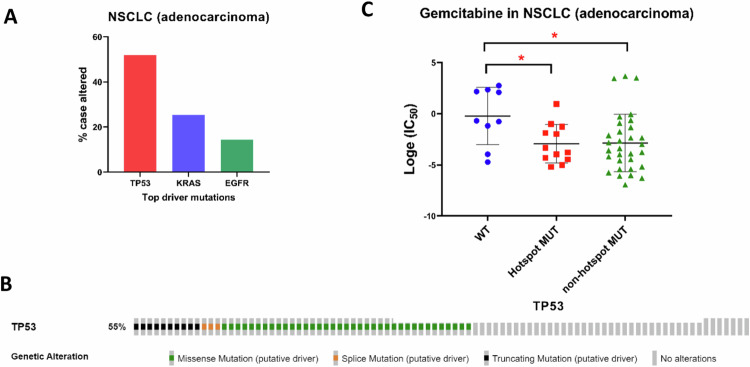
Gemcitabine response in NSCLC cells with *TP53* mutations. **A** The Cancer Genome Atlas (TCGA) database highlights *TP53* as one of the most frequently mutated genes in adenocarcinoma of non-small cell lung cancer (NSCLC). **B** Data extracted from the Cancer Genome Atlas (TCGA) database reveals the mutation frequency of *TP53* in NSCLC adenocarcinoma. *TP53* mutations encompass 55% of cases, including 40.7% missense mutations, 11% truncating mutations, and 3.3% splice mutations. **C** A scatter plot illustrates the half-maximal inhibitory concentration (IC50) values for gemcitabine treatment in NSCLC adenocarcinoma cell lines categorized as *TP53* wild-type (WT), harboring hotspot mutations, or non-hotspot mutant. The data suggests potential variations in gemcitabine sensitivity based on *TP53* mutational status.

As dissected from The Cancer Genome Atlas (TCGA) database, the *TP53* gene demonstrates the highest mutation frequency within NSCLC (Fig. 1A). It is noteworthy that *TP53* mutant patients constitute roughly 55% of NSCLC cases. Among these *TP53* mutations, missense mutations account for 40.7%, truncated mutations for 11%, and splice mutations for 3.3% (MSK, [15, 16]) (Fig. 1B). Notably, the most prevalent of these, missense mutations, can be classified into several functional categories according to their impact on the p53 protein. These categories encompass structural/conformational mutations, DNA-binding domain mutations, contact domain mutations, tetramerization domain mutations, and hotspot mutations. Hotspot mutations pertain to specific nucleotide alterations that occur at higher frequencies within *TP53*. These mutations tend to cluster in specific gene regions, leading to amino acid substitutions. Codons encoding amino acids R175, G245, R248, R249, R273, and R282 were classified as hotspot mutations in concordance with the IARC dataset [17], as elucidated in “Methods”. These mutations often associate with increased p53 malfunction and are recurrently observed across diverse cancer types.

Given the intricacy of hotspot mutations, our approach involved the classification of cell lines into those with hotspot mutant and non-hotspot mutant characteristics, as facilitated by the GDSC database. Our analysis indicated that gemcitabine possesses considerable potential as a therapeutic agent against lung adenocarcinoma cell lines harboring *TP53* mutations. In comparison to *TP53* wild-type cell lines, both *TP53* hotspot mutant and non-hotspot mutant cell lines exhibited heightened sensitivity to gemcitabine, yielding *P* values of 0.028 and 0.025, respectively (Fig. 1C).

### Correlation between cellular prediction and clinical response

**Fig. 2:**
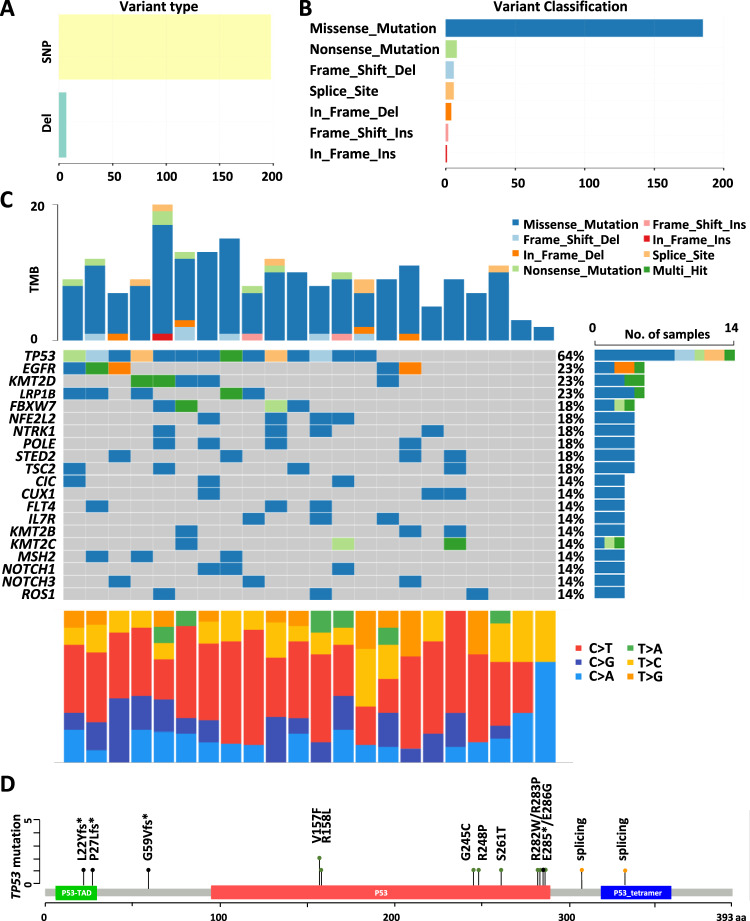
Mutational profiles of NSCLC patients from Taiwan. Patients who received first-line treatment were enrolled at Taipei Veterans General Hospital and their genomic alterations were analyzed using targeted re-resequencing. **A**, **B** Show the variant type and classification. **C** Depicts the top 20 gene mutation profiles for each patient. **D** Represents the positions of *TP53* mutations in NSCLC patients from Taiwan.

In a bid to establish a correlation between cellular prediction and clinical response, we conducted sequencing of 275 commonly implicated cancer-related genes across 39 patients. Among them, 22 patients received first-line gemcitabine while 17 patients received later-line gemcitabine. The comprehensive gene mutation profiles of the top 20 genes for each patient are visually presented in Fig. 2.

From the array of genetic alterations captured by our approach, single-nucleotide variations, and deletions emerged as the predominant mutation types (Fig. 2A). Functionally, the most noteworthy alterations encompassed missense, nonsense, frame-shift, and splice site mutations (Fig. 2B and Supplement Table 2). In alignment with TCGA datasets, *TP53* stood out with the highest overall mutation frequency in our cohort, being present in 14 out of 22 patients (64%). A total of 15 distinct *TP53* mutations were detected (Fig. 2C and Supplement Table 3). These *TP53* mutations comprised nonsynonymous changes (9/15, 60%), frameshift deletions (3/15, 20%), splice site mutations (2/15, 13.3%), and nonsense mutations (1/15, 6.7%) (Supplement Table 3). The positions and types of mutations within the *TP53* gene locus are visually depicted via the lollipop diagram in Fig. 2D.

**Fig. 3:**
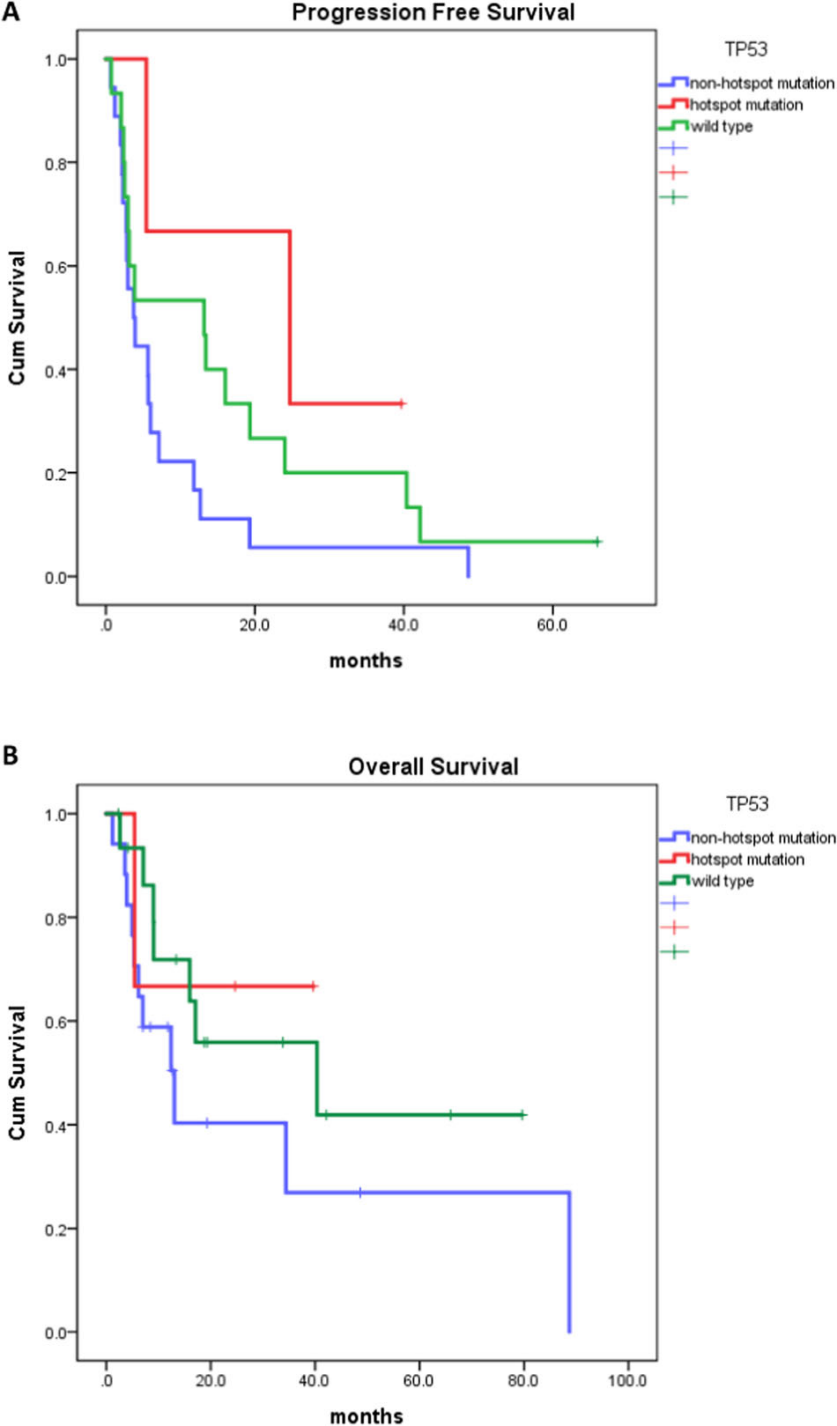
Progression-free survival (PFS) and overall survival (OS) of NSCLC patients treated with gemcitabine. **A** The PFS graph indicates a trend towards longer progression-free survival in patients with *TP53* hotspot mutations (*n* = 3) compared to other mutation groups. **B** The overall survival after receiving gemcitabine appears to be longer in the *TP53* hotspot mutation group, although the difference is not statistically significant (*P* value: 0.374) when compared to the *TP53* non-hotspot mutation group (*n* = 18) and the *TP53* wild-type group (*n* = 15).

Subsequently, our patients were classified into three groups, as detailed earlier: the *TP53* hotspot mutant group, the *TP53* non-hotspot mutant group, and the *TP53* wild-type group. In terms of gemcitabine response rate, the *TP53* hotspot mutation group exhibited a higher tendency in comparison to the *TP53* non-hotspot and *TP53* wild-type groups (100%, 41.7%, 35.7%, *P* value: 0.111). Notably, gemcitabine response was less favorable among NSCLC patients harboring non-hotspot *TP53* mutations (Table 1 and Fig. 3). Moreover, trends were observed in progression-free survival (PFS) and overall survival (OS) subsequent to gemcitabine treatment, with patients harboring hotspot mutations displaying the most favorable outcomes, followed by the *TP53* wild-type group and the non-hotspot *TP53* mutation group. However, these differences did not reach statistical significance due to the limited samples (PFS: 23.2 ± 8.1 months, 10.5 ± 3.1 months, 5.1 ± 1.4 months). Our findings suggest the potential utility of hotspot *TP53* mutations as biomarkers for gemcitabine treatment in NSCLC.

**Table 1:** Gemcitabine treatment response in patients.

TP53	Hotspot mutation (*n* = 3)	Not-hotspot mutation (*n* = 36)	Wild (*n* = 27)	*P* value
Response Rate (%)	3 (100%)	15 (41.7%)	10 (35.7%)	0.111
PFS (months)	23.2 ± 8.1	10.5 ± 3.1	5.1 ± 1.4	0.085
OS (months)	73.4 ± 27.8	38.4 ± 8.4	32.1 ± 9.1	0.259

This table summarizes the response of patients who received gemcitabine treatment. The treatment response is categorized based on different TP53 mutation groups.

### Sensitivity of *TP53* hotspot and null mutated cells to gemcitabine

To ascertain the susceptibility of *TP53* hotspot mutations to gemcitabine, we introduced various *TP53* hotspot mutations and wild-type variants into the H1299 *TP53*-Null cell line. This allowed us to evaluate cellular viability in response to gemcitabine treatment. Notably, *TP53* mutations frequently occur within the DNA-binding domain, with specific codons (175, 245, 248, 249, 273, and 282) consistently identified as “hotspot” codons. These hotspot mutations are divided into two distinct groups: those influencing p53 protein conformation (175, 245, 249, 282) and those impacting DNA contact domain (248, 273) [18]. Moreover, the majority of hotspot mutations result in the loss of p53 wild-type functions (loss-of-functions, LOF), while certain mutations acquire new oncogenic activities independently of p53 wild-type (gain-of-function, GOF).

Given the intricate nature of p53 mutations, we selected three distinct hotspot mutations for investigation into their effects on NSCLC cells under gemcitabine treatment. Specifically, the two most prevalent hotspot mutations, R248W and R273H, located in the DNA contact domain, along with R175H, which disrupts protein conformation in p53, were overexpressed in the H1299 *TP53*-Null cell line using lentiviral transfection. The efficacy of expression was verified via western blot analysis (Fig. S1).

**Fig. 4:**
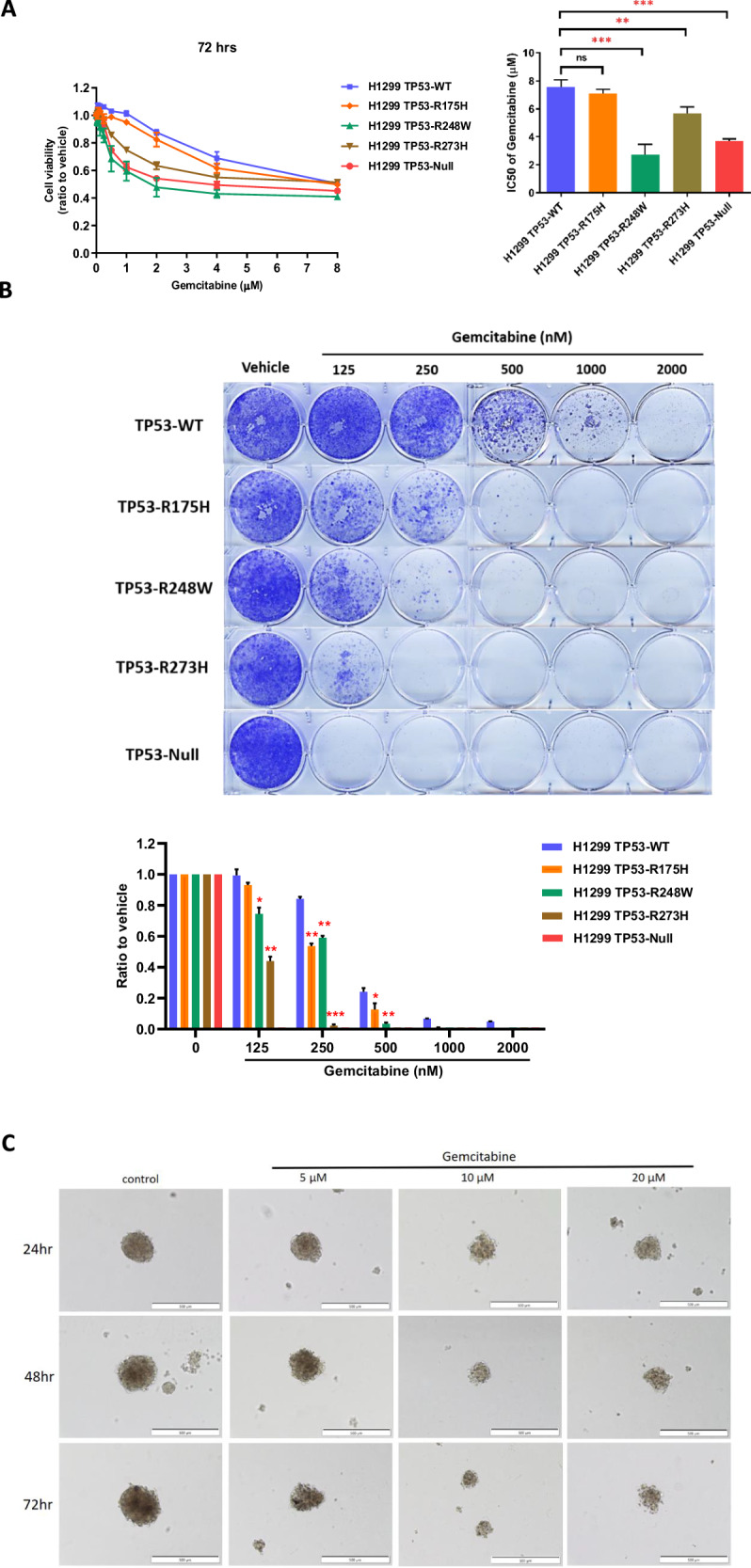
Sensitivity of *TP53* hotspot mutations and *TP53*-deficient cells to gemcitabine. **A** Cell viability (left) and according IC_50_ values (right) for different *TP53* hotspot mutations overexpressed in H1299 cells, as determined by the SRB assay, were significantly lower than those for H1299 *TP53* wild-type (WT) cells. **B** The cell viability of H1299 *TP53* hotspot mutant cells and *TP53*-Null cells over a period of 10 days was lower than that of H1299 *TP53-*WT cells. Notably, the R248W mutation, which was found in patients with partial responsiveness to gemcitabine (Table 1), exhibited the highest sensitivity to gemcitabine treatment among the hotspot mutations. The data presented in the graphs are shown as mean ± standard deviation (SD). Statistical significance is denoted as follows: *P < 0.05, **P < 0.01, ***P < 0.001, ****P < 0.0001 compared to control conditions. **C** Drug effect on gemcitabine treated H1299 spheroid. H1299 cells were seeded on the R^3^CE culture plate in the density of 800 cells/well for 7 days. While spheroids formed, gemcitabine was added to the medium for 1 to 3 days. To investigate the effects of gemcitabine on H1299 spheroids, three different concentrations were used, 5 μM, 10 μM and 20 μM. Cell morphology was observed with a phase contrast microscope with the ×10 objective. Scale bar: 500 μm.

Comparative analysis revealed that when compared to H1299 *TP53*-wild-type (WT) cells, the viability of cells carrying hotspot mutations, including *TP53*-R248W, R273H, and R175H, was notably lower. Of significance, *TP53*-R248W exhibited an approximately threefold lower IC50 (Fig. 4A). Remarkably, this specific hotspot mutation codon was also detected in an NSCLC patient who displayed a partial response to gemcitabine treatment in our patient sequence data.

However, in terms of cytotoxicity following 72 h, there was no significant difference in gemcitabine’s effect on H1299 *TP53*-R175H cells compared to and H1299 *TP53*-WT cells, hinting at varied impacts of different *TP53* mutations on cell growth regulation. Analogous outcomes were observed in colony formation over a 10-day period under gemcitabine treatmB). Notably, H1299 *TP53*-WT cells exhibited the highest resistance to treatment. Conversely, H1299 cells with other *TP53* hotspot mutations showcased significantly greater sensitivity to treatment than H1299 *TP53*-WT cells. Intriguingly, H1299 *TP53*-Null cells displayed the highest sensitivity to treatment, with minimal colony formation evident even at the lowest dose of treatment.

Collectively, these findings highlight that hotspot mutations, including *TP53*-R248W, R273H, and R175H, exhibit heightened sensitivity to gemcitabine compared to *TP53*-WT cells. Furthermore, it is evident that *TP53*-Null cells manifest the greatest sensitivity to gemcitabine treatment. Furthermore, due to the closer resemblance of 3D models to the in vivo environment, we have also established a 3D model of H1299 cells and conducted experiments with gemcitabine treatment. As shown in Fig. 4C, there are significant morphological differences in cells after drug treatment.

### Knockdown *TP53* enhanced the cellular sensitivity to gemcitabine

Given the pronounced susceptibility of *TP53*-Null mutated cells to gemcitabine, we postulate that the loss of p53 functions contributes to the higher sensitivity observed in *TP53* null and hotspot mutated H1299 cells. These cells are functionally devoid of p53, in accordance with the International Agency for Research on Cancer (IARC) *TP53* database. To validate this hypothesis, we embarked on *TP53* knockdown experiments using the A549, a human NSCLC cell line. Through lentiviral infection, we successfully established stable A549 *TP53*-knockdown lines. Subsequent western blotting confirmed the successful knockdown of *TP53* in these lines (Fig. S1A).

**Fig. 5:**
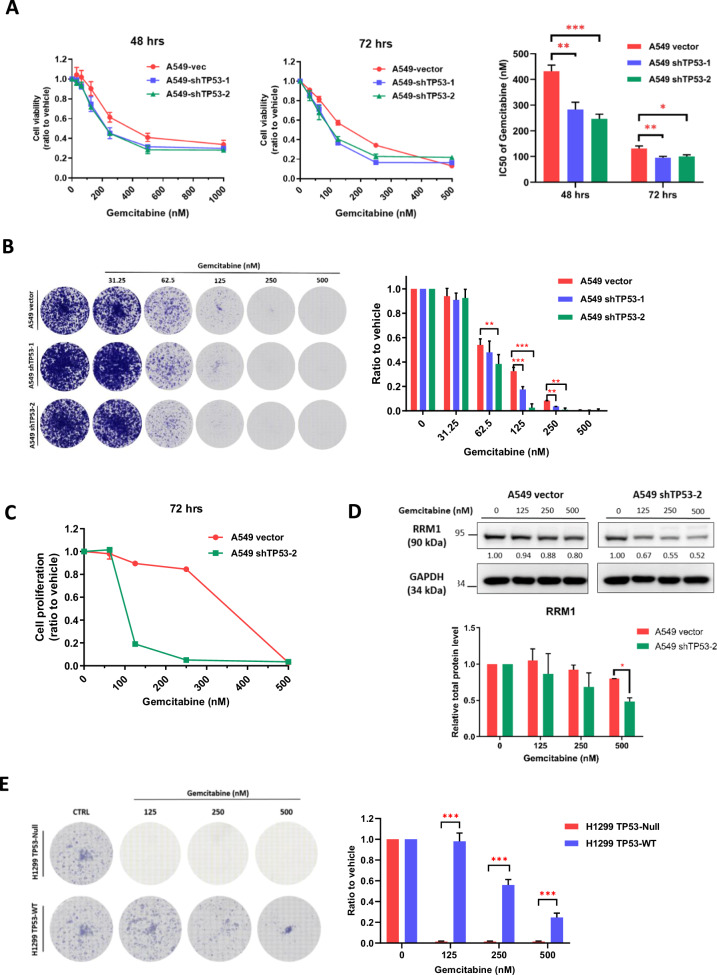
Sensitivity of *TP53* knockdown cells to gemcitabine treatment. **A** *TP53* knockdown in A549 cells using different short-hairpin RNAs (sh*TP53*-1 and sh*TP53*-2) resulted in significantly lower IC50 values compared to the non-treatment (control) in the sulforhodamine cytotoxicity assay. **B** The clonogenic assay showed a dramatic decrease in cell viability in *TP53* knockdown cells compared to control cells. **C** The cell proliferation assay using BrdU labeling within 4 h showed that A549 *TP53* wild-type (WT) cells were more resistant than A549 *TP53* knockdown cells after 72 h of gemcitabine treatment in a 96-well plate. **D** Western blot analysis of RRM1, a gemcitabine target protein, showed variable protein levels (left) in A549 vector and *TP53* knockdown cells exposed to different doses of gemcitabine for 72 h. Relative quantification analysis (right) was normalized by GAPDH and corrected by the control. **E** H1299 *TP53*-Null cells exhibited a dramatic decrease in cell viability after 7 days of gemcitabine treatment, in contrast to H1299 *TP53*-WT cells. Data in the graphs are presented as mean ± standard deviation (SD). Statistical significance is denoted as follows: *P < 0.05, **P < 0.01, ***P < 0.001, ****P < 0.0001 compared to control conditions.

Our findings revealed that A549 *TP53*-knockdown cells displayed greater sensitivity to gemcitabine compared to *TP53* wild-type cells (Fig. 5A). Furthermore, gemcitabine treatment induced a more pronounced reduction in colony formation ability among *TP53*-knockdown cells in comparison to *TP53* wild-type cells (Fig. 5B). Substantiating these observations, BrdU cell proliferation assays demonstrated that gemcitabine more effectively inhibited the proliferation of *TP53* knockdown cells than that of *TP53* wild-type cells (Fig. 5C). In addition, our data indicated a correlation between gemcitabine resistance and RRM1 expression [19]. RRM1 is a component of ribonucleoside-diphosphate reductase, a crucial enzyme for deoxyribonucleotide synthesis [20]. The diphosphorylated form of gemcitabine inhibits this enzyme, thus reducing nucleotide production. Notably, RRM1 expression in A549 *TP53*-knockdown cells decreased in a dose-dependent manner upon gemcitabine treatment, while no such change was observed in A549 *TP53* wild-type cells (Fig. 5D).

Similarly, H1299 *TP53*-Null cells exhibited significantly lower cell viability and diminished colony formation capacity within 5 days in response to dose-dependent gemcitabine treatment. In contrast, H1299 *TP53*-WT cells demonstrated a tendency toward increased resistance to gemcitabine treatment, particularly at lower doses (Fig. 5E).

Collectively, our data underscore the crucial role of p53 functional deficiency in sensitizing NSCLC cells to gemcitabine treatment. This insight provides valuable implications for understanding the intricate interplay between p53 status and gemcitabine response in the context of NSCLC.

### DNA damage accumulation induced by p53 ablation in NSCLC cells upon gemcitabine treatment

**Fig. 6:**
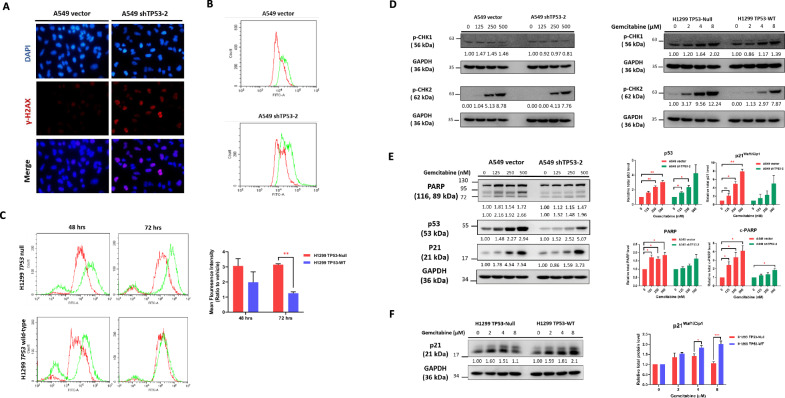
DNA damage and cell cycle responses to gemcitabine treatment in *TP53* knockdown vs. wild-type A549 cells. **A** Immunofluorescence staining for γ-H2AX (red color, 568 nm) in A549 cells after 125 nM gemcitabine treatment. Nuclei were counterstained with DAPI (blue color, 461 nm). **B** Flow cytometry analysis of γ-H2AX signal, indicating DNA damage levels. *TP53* knockdown cells displayed higher γ-H2AX expression, signifying increased DNA damage under gemcitabine treatment. **C** Recovery of DNA damage in H1299 cells, measured by mean fluorescence intensity (MFI) of γ-H2AX antibody staining using FACS. Cells were treated with 4 µM gemcitabine for 48 and 72 h. **D** Western blot analysis of cell cycle checkpoint kinases CHK1 and CHK2 in response to DNA damage after 72-h gemcitabine treatment at different concentrations in A549 cells. **E** Similar Western blot analysis of p-CHK1 and p-CHK2 in H1299 cells after 72 h gemcitabine treatment at different concentrations. **F** Western blot analysis of cell cycle arrest (p53, p21) and DNA repair (PARP) markers after 72-hour gemcitabine treatment at different doses in A549 cells. P53 overexpressed H1299 cells demonstrated dose-dependent induction of p21 expression under gemcitabine treatment. Data in the graphs are presented as mean ± standard deviation (SD). Statistical significance is indicated as follows: *P< 0.05, **P < 0.01, ***P < 0.001, ****P < 0.0001 compared to control conditions.

Gemcitabine, a DNA damage-inducing agent, generates triphosphate adducts that lead to masked chain termination during interphase, ultimately resulting in double-strand DNA breaks. To comprehensively understand the enhanced sensitivity of p53-deficient cells to gemcitabine, we conducted a series of investigations. Our first approach involved immunofluorescence staining to detect γ-H2AX (S139), a marker for permanent DNA damage, followed by quantification of γ-H2AX (S319) levels using flow cytometry. Notably, both *TP53*-deficient A549 and H1299 cells exhibited elevated γ-H2AX (S319) signals (Fig. 6A–C). Conversely, *TP53* wild-type overexpressed cells displayed diminishing γ-H2AX (S319) signals in a dose and time-dependent manner, indicative of increased DNA damage repair in these cells.

In the context of DNA damage response pathways, two canonical signaling cascades are pivotal: the ATM-CHK2-p53 pathway for double-strand breaks and the ATR-CHK1 pathway for single-strand breaks. Our western blot findings indicated a key role for ATM-CHK2-p53 signaling in the response to gemcitabine-induced DNA damage. Notably, levels of phosphorylated CHK2 (p-CHK2 S19) markedly increased in both H1299 and A549 cells in a dose-dependent manner. Notably, H1299 *TP53*-Null cells exhibited higher levels of p-CHK2, reflecting a feedback response due to the incapacity to induce p53 (Fig. 6D).

Conversely, there was negligible change in p-CHK1 (S317) levels in A549 cells, while a slight increase was noted in H1299 cells (Fig. 6D). In addition, the DNA repair marker PARP demonstrated significant induction in p53-WT cells compared to p53 knockdown cells (Fig. 6E). Furthermore, the protein expression levels of p53 and its downstream effector, p21, exhibited substantial upregulation in A549 cells. Exogenous overexpression of p53 in H1299 cells similarly led to a dose-dependent increase in p21 expression upon gemcitabine treatment (Fig. 6F). Of note, the hyperphosphorylated upper band of p21 in H1299 cells was observed. Collectively, our data provide compelling evidence that the ATM-CHK2-p53-p21 signaling pathway plays a pivotal role in the DNA damage response induced by gemcitabine, facilitating DNA repair.

### Induction of G2/M cell cycle arrest and cell death by p53 downregulation after gemcitabine-induced DNA damage

**Fig. 7:**
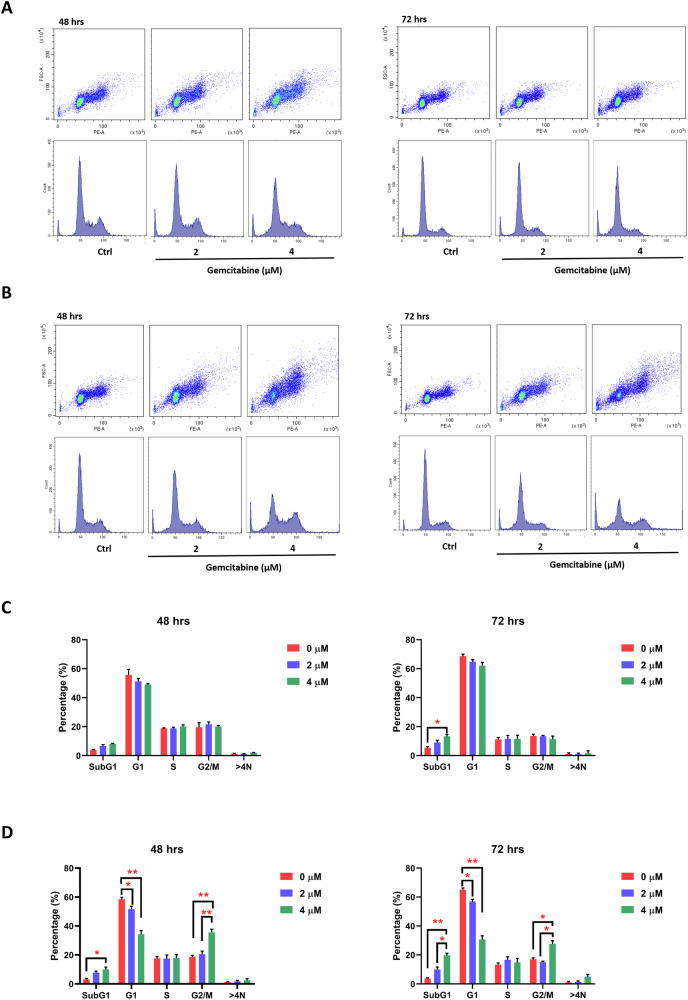
Gemcitabine-induced G2/M arrest and apoptosis in H1299 *TP53*-null cells. This figure illustrates the impact of gemcitabine treatment on cell cycle progression and apoptosis in H1299 *TP53*-Null cells. Cell cycle analysis by flow cytometry is performed on both H1299 *TP53* wild-type (WT) (**A**) and *TP53*-Null cells (**B**) after 48 h and 72 h of gemcitabine treatment. **C**, **D** The graphs display the population distribution among different cell cycle phases in H1299 *TP53*-WT cells and *TP53*-Null cells under gemcitabine treatment for 48 and 72 h, respectively. The data are presented as mean ± standard deviation (SD). The figure demonstrates that gemcitabine treatment induces G2/M arrest and apoptosis in H1299 *TP53*-Null cells, as reflected in the altered cell cycle distribution and the population of cells in various phases. The observed effects of gemcitabine treatment are compared between H1299 *TP53*-WT and *TP53*-Null cells. Statistically significant differences are indicated by asterisks in the graphs.

To assess whether the accumulated DNA damage perturbed cell cycle progression and leads to cancer cell death, which attribute to the diminished cell viability and reduced colony formation observed in *TP53*-deficient cells following gemcitabine-induced-DNA damage, we evaluated cell cycle at 48 and 72 h in H1299 *TP53*-Null and overexpressed *TP53*-WT cells. Intriguingly, in *TP53*-WT cells, the proportion of cells in G0/G1, S, G2/M phase unchanged in a time-dependent manner as shown in Fig. 7A, C. However, an elevated G2/M phase proportion of cells was observed in H1299 *TP53*-Null cells in a dose-dependent manner, alongside a considerable increase in the sub G1 population compared to *TP53*-WT cells (Fig. 7B, D). Cyclin B1, which typically degrades before mitotic exit, highly accumulated in *TP53*-null cells, indicating mitotic arrest and contributing to mitotic defects. Simultaneously, phosphorylation of CDK1 at T161 was reduced in *TP53*-null cells but not in *TP53*-WT cells (Fig. S3A). Furthermore, there was a significant increase in the sub-G1 cell population in a time-dependent manner indicating cells subsequently go to apoptosis pathway. For *TP53*-WT cells, apoptosis cell population only increased at higher dose after 72 h treatment. Besides, an increase in the sub G2/M population ( > 4 N) was observed in *TP53*-Null cells, although it was not statistically significant, suggesting that few cells experienced mitotic failure, possibly leading to cell death pathways. In A549 *TP53*-knockdown cells, we also observed the induction of multinucleated cells after 96 h under treatment of low-dose gemcitabine by immunostaining with antibodies against pericentrin, α-tubulin, and DAPI (Fig. S2). Noticeably, mitotic kinase Aurora-A was downregulated in *TP53*-null cells but increased in wild-type cells (Fig. S3A).

Collectively, these findings underscore that the disruption of p53 function, in conjunction with gemcitabine treatment, leads to cell cycle arrest at the G2/M phase or results in mitotic defects, ultimately driving cells to undergo apoptosis.

### Potential mechanism of *TP53*-mutation-mediated sensitivity of gemcitabine in NSCLC

**Fig. 8:**
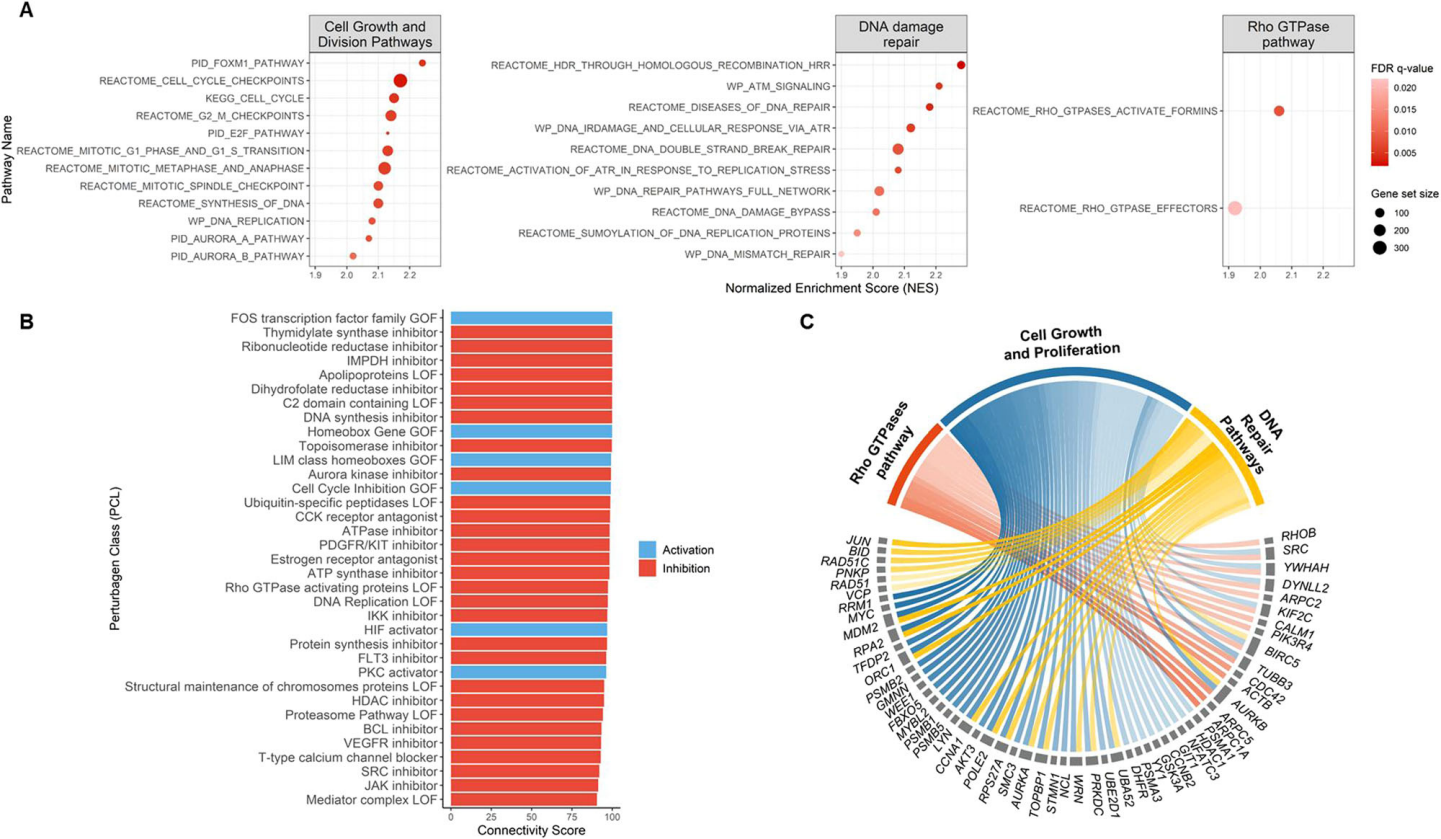
In silico analysis suggests the underlying mechanism of gemcitabine sensitivity in *TP53*-mutated NSCLC. **A** Gene Set Enrichment Analysis (GSEA) of the TCGA-LUAD dataset comparing *TP53*-mutant and wild-type patients. Enriched pathways are categorized into three major groups: cell growth and division, DNA damage repair, and Rho GTPase signaling. Dot colors represent the normalized enrichment score (NES), while dot sizes indicate the number of genes in each gene set. **B** Perturbational classes (PCLs) strongly linked to gemcitabine, retrieved via the CLUE platform. Red indicates inhibitory PCLs, while blue indicates activating PCLs. **C** Gene knockdown (KD) signatures highly associated with gemcitabine, also retrieved via CLUE. In the chord diagram, chords connect genes to their corresponding pathways, as defined in (**A**). Chord transparency reflects the CLUE connectivity score, with higher visibility indicating stronger similarity between gemcitabine treatment and gene KD effects. Connections with a connectivity score >90 are considered strong.

To elucidate the molecular signature associated with *TP53* mutations in LUAD, we analyzed the transcriptomic data from the TCGA-LUAD dataset. Patients were categorized into *TP53*-mutant and wild-type groups, and followed by gene set enrichment analysis (GSEA) to identify differentially regulated pathways. The data revealed that several classical pathways significantly upregulated in *TP53*-mutant LUAD patients were inhibited by gemcitabine, based on transcriptomic data from the CLUE platform. Specifically, pathways related to cell growth, division, and DNA damage response were upregulated in *TP53*-mutant patients (Fig. 8A). Gemcitabine treatment showed similarities to several perturbation classes (PCLs) that inhibit these processes, including structural maintenance of chromosomes protein loss-of-function (LOF), DNA replication LOF, cell cycle inhibition (gain-of-function (GOF)), aurora kinase inhibitors, topoisomerase inhibitors, DNA synthesis inhibitors, and ribonucleotide reductase inhibitors [21–25]. Furthermore, gemcitabine demonstrated connections to perturbations targeting DNA damage response, such as topoisomerase inhibitors, DNA replication LOF, DNA synthesis inhibitors, ribonucleotide reductase inhibitors, and thymidylate synthase inhibitors, suggesting its ability to disrupt DNA repair mechanisms (Fig. 8B).

In addition to these classical pathways, the analysis revealed significant upregulation of the Rho GTPase signaling pathway in *TP53*-mutant patients, which regulates cytoskeletal dynamics, cell migration, and cell cycle progression [25]. Simultaneously, gemcitabine treatment showed similarities to Rho GTPase-activating protein LOF, highlighting a novel aspect of its mechanism of action.

Furthermore, gemcitabine was associated with inhibitors of cell proliferation, metastasis, and angiogenesis, including JAK inhibitors, SRC inhibitors, BCL inhibitors, proteasome pathway LOF, HDAC inhibitors, IKK inhibitors, and VEGFR inhibitors [26, 27]. These connections underscore gemcitabine’s potential role in modulating critical cancer-related pathways. Finally, the connectivity profile from the CLUE platform revealed several gene knockdowns producing effects similar to gemcitabine treatment (Fig. 8C). These genes are involved in the pathways upregulated in *TP53*-mutant LUAD patients, suggesting potential molecular targets through which gemcitabine modulates these pathways.

### Clinical applications and future goals

**Fig. 9:**
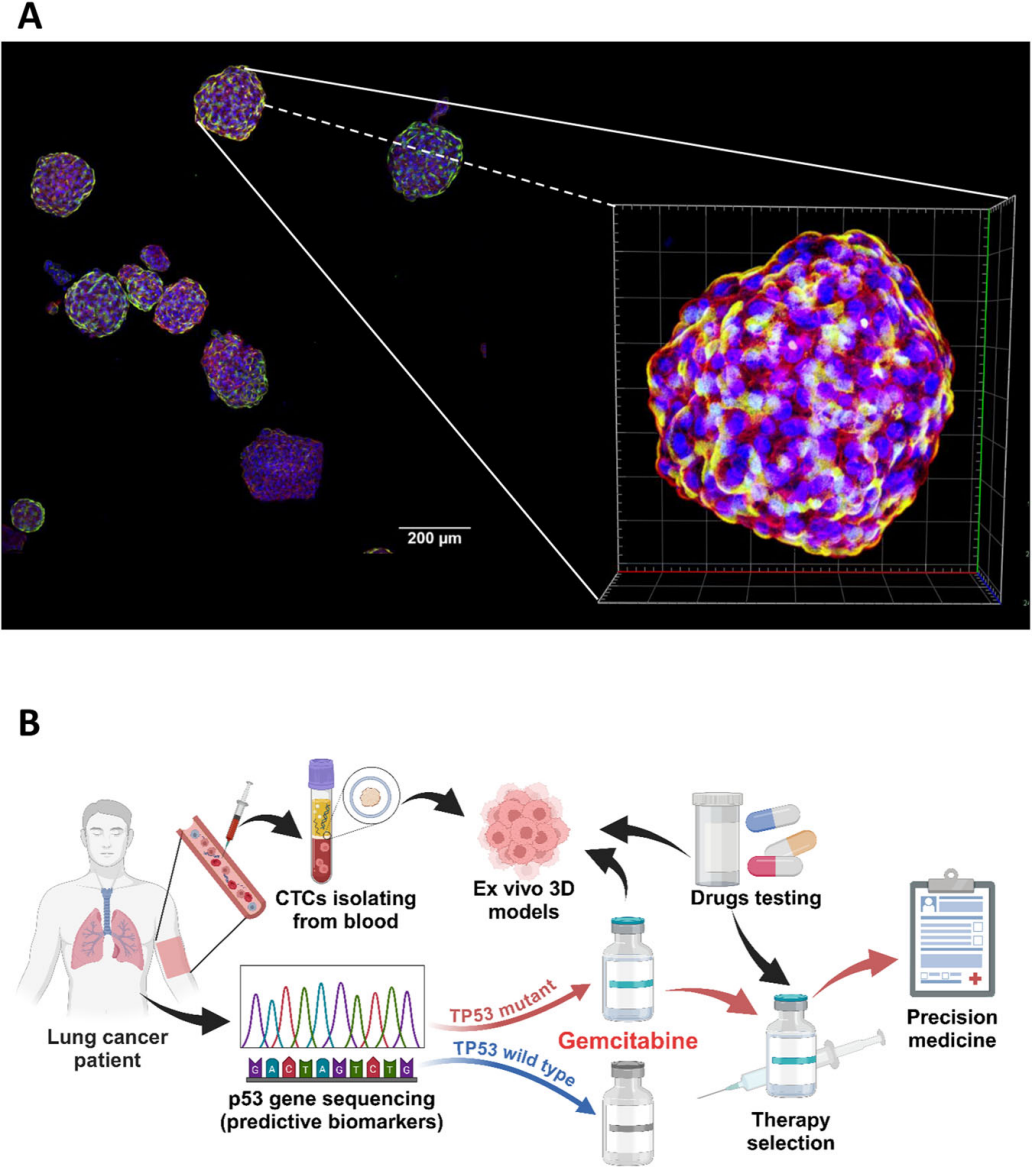
A schematic illustration of drug testing using 3D models. **A** Immunofluorescence staining of A549 spheroids. The A549 cells were seeded onto R^3^CE culture plate and cultured for a duration of 7 days. Confocal microscopy was employed of the spheroids with a 20X object. The spheroids were stained with CD44 (labeled with Alexa Fluor™ 488), Alexa Fluor™ 568 Phalloidin, and Hoechst 33342. **B** Process Diagram of Future Goals. Sequencing the genes of cancer patients and selecting treatment options for those with *TP53*-related hotspot mutations for gemcitabine chemotherapy.

Based on the aforementioned, there is an increasing trend in gemcitabine sensitivity associated with p53 gene mutation. We aim to apply this conclusion to cancer patients in the clinical setting. By establishing 3D models, we can mimic the tumor environment within the human body without the need for animal or human trials (Fig. 9A). Through genetic sequencing, we can identify patients who are better suited for gemcitabine chemotherapy, particularly those with p53 mutations or deletions. This approach is expected to reduce lengthy and erroneous drug trials, achieve a more efficient cancer suppression effect, and contribute to the goal of precision medicine (Fig. 9B).

## Discussion

This study sheds light on the response of lung cancer cells and lung cancer patients with *TP53* hotspot mutations to gemcitabine treatment in comparison to those with *TP53* wild-type mutations. A meta-analysis has previously indicated that concurrent *TP53* mutations result in a poorer prognosis when employing targeted therapies [28]. Notably, recent research has shown that the combination of chemotherapies with targeted therapy yields increased survival rates in contrast to targeted therapies alone [29]. Despite this, the optimal chemotherapy to be paired with targeted therapies remains undetermined. The present study may suggest that gemcitabine could be an effective regimen for treating NSCLC patients with *TP53* hotspot mutations. Further clinical trials are essential to validate the efficacy of gemcitabine against *TP53* hotspot mutations.

P53 is a well-known regulator of the cell cycle in normal cells, playing a crucial regulatory role in various cell signaling pathways such as the cell cycle, intrinsic cell apoptosis, and DNA damage repair, all pivotal for maintaining genomic stability [30]. It can arrest the cell cycle at the G1/S, activating the process of DNA repair. It can also initiate apoptosis on irreversible DNA damage [31, 32]. Thus, *TP53* is viewed as a tumor suppressor. The majority of mutations of *TP53* in human cancers are missense mutations [33]. Only a single nucleotide alternate with another one leading to the amino acid substitutions. The p53 protein contains 393 residues and forms several functional domains, including the transactivation domain, proline-rich region, core DNA-binding domain, tetramerization domain, and the negative regulatory domain [34]. The cancer-associated *TP53* mutations are diverse in their locations and affect the thermodynamic stability of the p53 protein. In addition, the majority of missense mutations cause the loss of DNA-binding function and therefore the transcription function to activate the targeted downstream genes. Of the mutations in this domain, about 30% fall within 6 hotspot residues (R175, G245, R248, R249, R273, and R282) and are frequently observed in various cancers [35]. The functional consequences of these alternations encompass loss-of-function, dominant-negative effects, and gain-of-function alterations [36]. Hence, this study sought to establish a *TP53* mutant spectrum that might influence the efficacy of gemcitabine treatment. Our patient’s response data to gemcitabine treatment suggests a potential for higher response rates in patients with hotspot mutations. We began with the most prevalent mutations (R273H, R175H, and R248W) in NSCLC. These mutations fall within the top 30% of hotspot residues found in human cancers and are commonly associated with a loss of transactivation function. Nevertheless, some studies have proposed that they may also confer gain-of-function properties contributing to cancer progression [37–41].

An interesting observation is the substantial reduction in sensitivity in H1299 *TP53*-Null cells and H1299 *TP53*-R248W, accompanied by a minor sensitivity decrease in H1299 *TP53*-R273H, compared to H1299 *TP53*-WT cells. Conversely, H1299 *TP53*-R175H exhibited a sensitivity profile similar to that of H1299 *TP53*-WT. Although all these hotspot mutations lead to a loss of transactivation function, distinct mutation patterns could yield differing effects on gemcitabine treatment responses. The enhanced susceptibility of these cells to gemcitabine can be attributed to the disruption in p53 function, a conclusion that is further supported by our experiments involving A549 *TP53* knockdown cells (Fig. 5A–D).

Through in vitro validation using an NSCLC cell line model, we have shown that the downregulation of p53 correlates with heightened sensitivity to gemcitabine treatment. *TP53* wild-type cells exhibited lower levels of DNA damage relative to *TP53* knockdown cells due to reversible cell cycle arrest during the G1/S phases, allowing DNA break repair to transpire (Figs. 6C, S3C, and S4). Upon substantial or severe DNA damage, p53 activated the apoptosis pathway by inducing the cleavage of PARP-1. In addition, intrinsic pro-apoptotic caspases like caspase 9 and caspase 3 were triggered to mediate cell death signaling. Consequently, gemcitabine treatment induced notably greater DNA damage in *TP53*-knockdown cells compared to *TP53*-WT cell (Fig. 6A–C). Our findings further highlighted that the p53-mediated enhancement of p21 and PARP-1 prompted the initiation of cell cycle arrest, followed by DNA damage repair consequent to gemcitabine treatment (Fig. 6F, G). Upon prolonged treatment with higher gemcitabine doses, *TP53* wild-type A549 cells commenced recovery and colony formation under drug-free conditions. In contrast, *TP53* knockdown A549 cells ceased growth. These outcomes underscore a potential connection between the role of p53-mediated DNA damage repair and gemcitabine treatment resistance in NSCLC.

Our investigation of mitotic abnormality markers and associated pathways revealed that Aurora-A, a mitotic kinase, was downregulated in TP53-null cells but upregulated in TP53-WT cells. Cyclin B1, critical protein for mitotic progression, accumulated in TP53-null cells, indicating mitotic arrest and contributing to mitotic defects. In addition, phosphorylated CDK1 (T161) was reduced in TP53-null cells, potentially contributing to G2/M cell cycle arrest. Persistent γ-H2AX levels in TP53-null cells, in contrast to their decline in TP53-WT cells, underscore the sustained DNA damage response caused by p53 loss. Although the regulatory mechanisms governing these markers remain unclear, prior studies suggest a potential role for CHK2. Besides, gemcitabine treatment in TP53-knockdown cells resulted in reduced Rb protein levels, indicating a loss of G1/S phase control and increased susceptibility to DNA damage.

Building on these findings, we observed that the inability of TP53-null cells to repair DNA damage during replication caused by gemcitabine treatment led to their progression into mitosis. However, these cells became arrested at the G2/M phase due to dysregulation of the Cyclin B1/CDK1 complex, eventually leading to apoptosis. In addition, multinucleated cells were observed, likely due to the downregulation of mitotic kinases such as Aurora-A, which impaired chromosome segregation. This disruption might trigger alternative cell death pathways, including mitotic catastrophe [42, 43].

In order to further assess other possible mechanisms, our transcriptomic analysis of the TCGA-LUAD dataset revealed key molecular alterations associated with TP53 mutations in LUAD. By performing gene set enrichment analysis (GSEA), we identified several key pathways that were differentially regulated between TP53-mutant and wild-type LUAD patients (Fig. 8A). Complementing these findings, transcriptomic data from the CLUE platform demonstrated that gemcitabine treatment in cancer cell lines strongly connected to the inhibition of pathways upregulated in TP53-mutant LUAD patients (Fig. 8B). Notably, several classical pathways involved in the cell cycle, DNA damage response, and cell growth showed strong connectivity between gemcitabine and TP53-mutant patients. These findings align with the known roles of TP53 in regulating the cell cycle and DNA repair, suggesting that TP53 mutations may drive aberrant cellular processes that support tumorigenesis in NSCLC [44, 45]. In addition, this data also supports our hypothesis that gemcitabine induces DNA damage response and cell cycle arrest through the ATM-CHK2-p53 pathway. Interestingly, Rho GTPase signaling, which is crucial for regulating cytoskeletal dynamics, cell migration, and cell cycle progression, was significantly upregulated in TP53-mutant patients. A key finding from our analysis is the novel link between gemcitabine and the Rho GTPase signaling pathway, suggesting a potential mechanism through which gemcitabine may impact these cellular processes [46]. In cancer, the Rho GTPase pathway is frequently dysregulated, promoting enhanced cell proliferation, invasion, and metastasis. Targeting this pathway has emerged as a promising therapeutic strategy, as modulating it can disrupt cancer cell motility and invasiveness. In lung cancer, Rho GTPases, especially RhoA, Rac1, and Cdc42, play key roles in tumor progression and metastasis [46, 47]. Therefore, these data suggest that gemcitabine may potentially modulate the Rho GTPase pathway, contributing to its anticancer efficacy.

This study has several limitations. First, the number of patients with *TP53* mutations included in our study is limited. In addition, the majority of patients underwent gemcitabine treatment at a later stage of their therapy, which could potentially impact gemcitabine response in patients harboring *TP53* mutations. Second, given the intricate nature of *TP53* mutations and their variability, cell line models may not fully represent the genomic diversity of all patients. Thus, this study requires further extension to encompass various mutation groups. Third, the interaction between drugs and cancer cells within the tumor microenvironment could be another factor influencing drug response. As a result, our predictive biomarkers should be further validated in diverse models, particularly through patient tissue analyses. In future research, we intend to amass more patient samples subjected to gemcitabine treatment to clinically confirm the sensitivity and selectivity of the proposed biomarkers.

## Methods

### Dataset

The Genomics of Drug Sensitivity in Cancer (GDSC) database was employed as our data source, encompassing interactions involving 516 compounds and 310 genetic alterations across 73 NSCLC cell lines. The primary objective was to identify a putative drug. This was achieved by comparing IC50 values of diverse compounds against distinct cell lines.

### Cell culture

Human NSCLC cell lines, A549 and H1299, were procured from ATCC. A549 cells were cultured in RPMI-1640 medium supplemented with 10% fetal bovine serum, 1% l-glutamine, 1% nonessential amino acids, and 1% penicillin/streptomycin (PS) at 37 °C within a 5% CO_2_ atmosphere. Likewise, H1299 cells were cultured in RPMI-1640 medium supplemented with 10% fetal bovine serum and 1% PS, also maintained at 37 °C under a 5% CO_2_ environment.

### 3D spheroid culture

Human NSCLC cell lines, A549 and H1299 were seeded onto the R^3^CE (Acrocyte Therapeutics Inc., New Taipei City, Taiwan) 3D culture platform and supplied with complete medium cultured for 7–10 days. The 3D culturing condition was conducted according to the previous cell culturing methods. The cell morphology was observed using Olympus IX83 inverted microscope before and after drug treatments.

### Gemcitabine response evaluation

To assess the sensitivity of both A549 and H1299 cell lines to gemcitabine, the viability of *TP53*-knockdown and *TP53* wild-type A549 cells was initially examined via Sulforhodamine B (SRB) assays after administering gemcitabine for 48 and 72 h, respectively.

### *TP53* knockdown

A549 cells exhibit a moderate level of p53 protein expression. shRNAs were procured from the RNAi Core (Academia Sinica), used pLKO.1 vector and featured the ensuing sequences: non-splicing shRNA control, m*TP53* (shRNA-1), 5’-GTCCAGATGAAGCTCCCAGAA-3’; m*TP53* (shRNA-2), 5’-GAGGGATGTTTGGGAGATGTA-3’. The transfection of shRNAs into A549 cells was facilitated through lentivirus. Subsequent selection of stable clones was carried out using 0.2 µg/ml puromycin within a 48-h window, followed by sustained maintenance for subsequent experimental analyses. The efficiency of knockdown in these cells was subsequently evaluated through quantitative western blot analysis.

### *TP53* overexpression

Lentivirus containing either wild-type *TP53* or harboring different hotspot mutations, including R248W, R175H, and R273H, cloned into the PLAS2w.Pneo lentiviral transfer vector, was used to transfect H1299 cells, which are p53 null. Overexpression of the *TP53* wild-type or mutated proteins is driven by a CMV promoter. After infection with lentivirus containing these plasmids (MOI = 2), stable colonies were selected with the antibiotic G418 (800 µg/ml) within 10 days and confirmed using a luciferase assay and western blot.

### Cell viability assay

Quantification of cell viability was executed through Sulforhodamine B (SRB) assays. A549 and H1299 cells were seeded in 96-well plates with cell growth medium, allowing a 16-hour incubation at a density of 3000 cells/well. Subsequently, cells were exposed to varying doses of gemcitabine for durations of 48 and 72 h. Post-drug exposure, cell monolayers were fixed using 10% (wt/vol) trichloroacetic acid (TCA) for 24 h, followed by rinsing with ddH_2_O. The monolayers were then stained with a 0.1% (wt/vol) SRB solution (Sigma, St. Louis, MO, USA) for an hour and subsequently washed thrice with a 1% (vol/vol) acetic acid solution. The dye, binding to proteins, was dissolved in a 10 mM Tris base solution, allowing optical density (OD) measurements at 540 nm via a microplate reader. The cell survival ratio was calculated as Mean OD_treat_/Mean OD_control_.

### Colony formation assay

Cell survival was assessed via a colony formation assay involving different gemcitabine doses administered for 24 h. Following this, cells were cultivated for several days depending on different experiments, after which they were subjected to washing, fixation, and staining using Crystal Violet (Sigma). Capturing of colonies was facilitated by an Olympus IX-83 microscope, with triplicate samples taken for each experiment. Colonies were counted if they contained a minimum of 50 cells per colony.

### Flow cytometry analysis

Cells were initially fixed in 2% paraformaldehyde for a duration of 10 min, followed by subsequent fixation in 70% ethanol over an overnight period. Following fixation, the cells underwent staining with propidium iodide (PI) and antibodies after a 30-minute incubation with 5 µg/ml RNase A. The Cytoflex XL flow cytometer (Beckman Coulter) was employed for cell analysis, and the Cytoplex program was employed to quantify the cell cycle phases.

### Western blot analysis

In total, 3 × 10^5^ cells were seeded into 6-cm dishes and incubated at 37 °C with 5% CO_2_ overnight. Post-drug treatment, floating cells were collected via centrifugation, washed using phosphate-buffered saline (PBS), and then scraped to obtain the cell pellet. Lysis of cells and subsequent centrifugation were performed to obtain the supernatant. The protein concentration was determined via a Bradford protein assay (Bio-Rad). A total of 30 µg of protein was denatured by heating at 99.9 °C for 10 min. Next, 20 µL/well (comprising 30 µg of protein + lysis buffer + 4× dye) was loaded onto SDS polyacrylamide gel electrophoresis (SDS-PAGE).

Electro-transfer of proteins onto a 0.45-µm PVDF membrane (Millipore, catalog number: IPVH00010) was executed. Subsequently, membrane blocking was carried out with 5% skim milk (BD, catalog number: 232100, dissolved in TBST) for one hour at room temperature. This was following by an overnight incubation at 4 °C with primary antibodies (as detailed in Supplement Table 1). Secondary antibodies (anti-rabbit or anti-mouse from Jackson Lab) were utilized at dilutions of 1:5000 or 1:3000, followed by a 1-h incubation at room temperature. Detection was accomplished via strong or weak enhanced chemiluminescence (ECL), and resultant images were captured using the Luminescence Imaging system (Fuji LAS-4000). Uncrop western blot images are included in Fig. S5.

### Immunofluorescence staining

Following two washes with Tris-buffered saline (TBS), cells underwent fixation using 4% paraformaldehyde (PFA) for 10 min. The cells were then washed twice with TBS. Permeabilization was performed for 5 min exposure to ice-cold 0.5% Triton X100, followed by 20 min incubation in 3% BSA diluted in 1× TBS.

The immunolabeling procedure commenced with the addition of primary antibodies (γ-H2AX antibody at 1:300, α-tubulin at 1:300, or pericentrin at 1:300, as detailed in Supplement Table 1) overnight with 50 rpm shaking. Subsequent to the removal of the staining solution, TBS was used for cell washing. Introduction of a secondary antibody (Alexa Fluor 568 anti-rabbit antibody or Alexa Fluor 488 anti-mouse antibody) at a 1:300 concentration was carried out, allowing a 45 min incubation while safeguarding samples from light. Post-secondary antibody incubation, the stain solution was removed, and cells were rinsed with PBS.

A counterstain for cellular nuclei was executed through the addition of DAPI stain solution at a concentration of 1:2000, all within 5 min, with light protection measures in place. Following a final PBS wash, mounting liquid was utilized to secure coverslips onto slides. Imaging was accomplished via a confocal LS700 microscope at a ×63 magnification or an Olympus BX61 upright fluorescence microscope system at a ×40 magnification. All images were subsequently analyzed using ImageJ software.

### Connectivity Map/CLUE analysis

The Connectivity Map (CMap) platform (https://clue.io/) is a computational tool that uncovers connections between drugs, genes, and diseases by analyzing cellular responses [48]. Differentially expressed genes representing a specific biological state, particularly gemcitabine treatment, were used to query the database. The platform then compared the query to its signatures and generated a rank-ordered list based on similarity. Connectivity scores were calculated using three metrics: (1) a nominal p-value from the Kolmogorov-Smirnov enrichment statistic, (2) a false discovery rate (FDR) for multiple hypothesis correction, and (3) Tau (τ) to contextualize enrichment scores within the database. This analysis identified Perturbational Classes (PCLs) and knockdown (KD) profiles resembling gemcitabine’s treatment effects, providing mechanistic insights and therapeutic potential.

### Enrichment analysis for transcriptomic profile of lung adenocarcinoma (LUAD) patients

Transcriptomic profile of LUAD patients, normalized by Transcripts Per Million (TPM) were retrieved from TCGA dataset via TCGAbiolinks package. The patients were then divided into two groups, with and without *TP53* mutations, for gene set enrichment analysis (GSEA).

GSEA computed an enrichment score for each gene set and normalized it based on the set’s size. The enrichment scores can be positive or negative indicating whether the gene set is up or downregulated, respectively. This study employed the C2 gene set collection from the MSigDB database for the analysis. GSEA was performed using 1000 permutations with a phenotype permutation type.

### Clinical sample collection, DNA sequencing, and data processing

To corroborate our findings through clinical response validation, we collected specimens from advanced NSCLC patients who had undergone gemcitabine treatment at Taipei Veterans General Hospital between 2015 and 2018. Tumor specimens were acquired during surgical procedures and subsequently fixed using formalin, following established protocols.

For each tumor sample, a single roll of ten-micrometer FFPE sections was utilized for the extraction of genomic DNA and subsequent library preparation. Library preparation procedures were conducted employing the QIAseq Human Comprehensive 275 Cancer Panel (DHS-3501Z, QIAGEN), adhering to the manufacturer’s provided instructions. In essence, each sample involved 50 ng of genomic DNA, which underwent initial fragmentation, followed by end-repair and A-tailing. Prepared DNA fragments were subsequently ligated at their 5’ ends with sequencing-specific adapters integrating UMIs (Unique Molecular Index) and sample indexes.

Post target enrichment and library amplification, the DNA library templates were evaluated for quality utilizing the Agilent Bioanalyzer 2100. Upon confirming library quality, qualified libraries underwent sequencing through the NextSeq500 instrument (Illumina) with the high Output Kit V2 (300 cycles), adhering to the manufacturer’s guidelines.

The interpretation of variants within the sequencing data was executed by the GeneGlobe Data Analysis Center (QIAGEN) [49], Subsequent data processing steps encompassed the exclusion of single nucleotide polymorphism sites and the application of variant mutation frequency filters. Specifically, for *TP53* alterations, *TP53* hotspot mutations included R175, G245, R248, R249, R273, and R282. Given *TP53*’s role as a transcription activator, these mutations were categorized based on their impact on the transcriptional activity across eight distinct promoters. This classification was guided by the International Agency for Research on Cancer (IARC) dataset, which aggregates a range of data and information concerning cancer-related *TP53* alterations [17].

### Statistical analysis

All data are expressed as mean ± standard deviation (SD). Discrepancies between the experimental and control groups were assessed using a two-tailed Student’s *t* test. Fisher’s exact test was employed to analyze categorical variables. Calculation of progression-free survival and overall survival was conducted through the Kaplan–Meier method. Significance levels were indicated as follows: *P < 0.05, **P < 0.01, ***P < 0.001, and ****P < 0.0001.

## Supplementary information

sup figure

sup table 1-3

original data

## Data Availability

The datasets used and/or analyzed during the current study are available from the corresponding author on reasonable request.
