# Correction: Utilizing *TP53* hotspot mutations as effective predictors of gemcitabine treatment outcome in non-small-cell lung cancer

**DOI:** 10.1038/s41420-025-02400-4

**Published:** 2025-04-25

**Authors:** Yen-Han Tseng, Trieu Thi My Tran, Jinghua Tsai Chang, Yu-Tang Huang, Anh Thuc Nguyen, Ian Yi-Feng Chang, Yi-Tung Chen, Hao-Wen Hsieh, Yue-Li Juang, Peter Mu-Hsin Chang, Tzu-Yi Huang, Ying-Chih Chang, Yuh-Min Chen, Hsuan Liu, Chi-Ying F. Huang

**Affiliations:** 1 Department of Chest Medicine, Taipei Veterans General Hospital, Taipei, Taiwan; 2 Program in Molecular Medicine, College of Life Sciences, National Yang Ming Chiao Tung University, Taipei, Taiwan; 3 School of Medicine, College of Medicine, National Yang Ming Chiao Tung University, Taipei, Taiwan; 4 Institute of Biopharmaceutical Sciences, College of Pharmaceutical Sciences, National Yang Ming Chiao Tung University, Taipei, Taiwan; 5 Institute of Biomedical Sciences, Academia Sinica, Taipei, 11529 Taiwan; 6 Institute of Medicine, Chung Shan Medical University, 110, Sec.1, Chien-Kuo N. Road, Taichung City, 402306 Taiwan; 7 Biomedical Industry Ph.D. Program, College of Life Sciences, National Yang Ming Chiao Tung University, Taipei, 112304 Taiwan; 8 Program in Molecular Medicine, Taiwan International Graduate Program in Molecular Medicine, National Yang Ming Chiao Tung University and Academia Sinica, Taipei, Taiwan; 9 Molecular Medicine Research Center, Chang Gung University, Taoyuan, Taiwan; 10 Department of Neurosurgery, Lin-Kou Medical Center, Chang Gung Memorial Hospital, Taoyuan, Taiwan; 11 Institute of Clinical Medicine, School of Medicine, National Yang Ming Chiao Tung University, Taipei, Taiwan; 12 Institute of Biomedical Sciences, Mackay Medical College, New Taipei City, Taiwan; 13 Department of Oncology, Taipei Veterans General Hospital, Taipei, Taiwan; 14 Genomics Research Center, Academia Sinica, Taipei, 115 Taiwan; 15 Department of Chemical Engineering, Stanford University, Stanford, CA 94305 USA; 16 Department of Cell and Molecular Biology, College of Medicine, Chang Gung University, Taoyuan, Taiwan; 17 Graduate Institute of Biomedical Sciences, College of Medicine, Chang Gung University, Taoyuan, Taiwan; 18 Division of Hematology-Oncology, Department of Internal Medicine, Chang Gung Memorial Hospital at Linkou, Taoyuan, Taiwan; 19 Department of Biochemistry, School of Medicine, Kaohsiung Medical University, Kaohsiung, Taiwan; 20 Chong Hin Loon Memorial Cancer and Biotherapy Research Center, National Yang Ming Chiao Tung University, Taipei, Taiwan

**Keywords:** Cell death, Cancer epigenetics, Non-small-cell lung cancer

Correction to: *Cell Death Discovery* 10.1038/s41420-025-02300-7, published online 27 January 2025

In this article, the order of affiliations and numbering assigned to the authors was mistakenly misaligned. Furthermore, our school's English name has also been recently changed.

Yen-Han Tseng^1,2,3,21^, Trieu Thi My Tran^4,5,21^, Jinghua Tsai Chang^6^, Yu-Tang Huang^4,7^, Anh Thuc Nguyen^4,8^, Ian Yi‑Feng Chang^8,9^, Yi-Tung Chen^8^, Hao-Wen Hsieh^10^, Yue-Li Juang^11^, Peter Mu-Hsin Chang^2,4,12,13^, Tzu-Yi Huang^3^, Ying-Chih Chang^14,15^, Yuh-Min Chen^1,2,*^, Hsuan Liu^7,16,17,18,**^ and Chi-Ying F. Huang^4,19,20,***^

^1^Department of Chest Medicine, Taipei Veterans General Hospital, Taipei, Taiwan.

^2^School of Medicine, National Yang Ming Chiao Tung University, Taipei, Taiwan.

^3^Program in Molecular Medicine, School of Life Sciences, National Yang Ming Chiao Tung University, Taipei, Taiwan.

^4^Institute of Biopharmaceutical Sciences, College of Pharmaceutical Sciences, National Yang Ming Chiao Tung University, Taipei, Taiwan.

^5^Institute of Biomedical Sciences, Academia Sinica, Taipei, Taiwan.

^6^Institute of Medicine, Chung Shan Medical University, Taichung City, Taiwan.

^7^Biomedical Industry Ph.D. Program, College of Life Sciences, National Yang Ming Chiao Tung University, Taipei 112304, Taiwan.

^8^Taiwan National Graduate Program in Molecular Medicine, National Yang Ming Chiao Tung University and Academia Sinica, Taipei, Taiwan.

^9^Molecular Medicine Research Center, Chang Gung University, Taoyuan, Taiwan.

^10^Department of Neurosurgery, Lin-Kou Medical Center, Chang Gung Memorial Hospital, Taoyuan, Taiwan.

^11^Institute of Clinical Medicine, School of Medicine, National Yang Ming Chiao Tung University, Taipei, Taiwan.

^12^Institute of Biomedical Sciences, Mackay Medical College, New Taipei City, Taiwan.

^13^Department of Oncology, Taipei Veterans General Hospital, Taipei, Taiwan.

^14^Genomics Research Center, Academia Sinica, Taipei 115, Taiwan.

^15^Department of Chemical Engineering, Stanford University, Stanford, CA, USA.

^16^Department of Cell and Molecular Biology, College of Medicine, Chang Gung University, Taoyuan, Taiwan.

^17^Graduate Institute of Biomedical Sciences, College of Medicine, Chang Gung University, Taoyuan, Taiwan.

^18^Division of Hematology-Oncology, Department of Internal Medicine, Chang Gung Memorial Hospital at Linkou, Taoyuan, Taiwan.

^19^Department of Biochemistry, School of Medicine, Kaohsiung Medical University, Kaohsiung, Taiwan.

^20^Chong Hin Loon Memorial Cancer and Biotherapy Research Center, National Yang Ming Chiao Tung University, Taipei, Taiwan.

Should read:

Yen-Han Tseng^1,2,3#^, Trieu Thi My Tran^4,5#^, Jinghua Tsai Chang^6^, Yu-Tang Huang^4,7^, Anh Thuc Nguyen^4,8^, Ian Yi-Feng Chang^9,10^, Yi-Tung Chen^9^, Hao-Wen Hsieh^11^, Yue-Li Juang^12^, Peter Mu-Hsin Chang^3,4,13^, Tzu-Yi Huang^2^, Ying-Chih Chang^14,15^, Yuh-Min Chen^1,3*^, Hsuan Liu^9,16,17,18*^, Chi-Ying F. Huang^4,19,20*^

^1^Department of Chest Medicine, Taipei Veterans General Hospital, Taipei, Taiwan

^2^Program in Molecular Medicine, College of Life Sciences, National Yang Ming Chiao Tung University, Taipei, Taiwan

^3^School of Medicine, College of Medicine, National Yang Ming Chiao Tung University, Taipei, Taiwan

^4^Institute of Biopharmaceutical Sciences, College of Pharmaceutical Sciences, National Yang Ming Chiao Tung University, Taipei, Taiwan

^5^Institute of Biomedical Sciences, Academia Sinica, Taipei 11529, Taiwan.

^6^Institute of Medicine, Chung Shan Medical University, 110, Sec.1, Chien-Kuo N. Road, Taichung City 402306, Taiwan

^7^Biomedical Industry Ph.D. Program, College of Life Sciences, National Yang Ming Chiao Tung University, Taipei 112304, Taiwan

^8^Taiwan International Graduate Program in Molecular Medicine, National Yang Ming Chiao Tung University and Academia Sinica, Taipei, Taiwan

^9^Molecular Medicine Research Center, Chang Gung University, Taoyuan, Taiwan

^10^Department of Neurosurgery, Lin-Kou Medical Center, Chang Gung Memorial Hospital, Taoyuan, Taiwan

^11^Institute of Clinical Medicine, School of Medicine, National Yang Ming Chiao Tung University, Taipei, Taiwan

^12^Institute of Biomedical Sciences, Mackay Medical College, New Taipei City, Taiwan

^13^Department of Oncology, Taipei Veterans General Hospital, Taipei, Taiwan

^14^Acrocyte Therapeutics, Inc. 12-8F, 99, Xintai 5th Rd, Xizhi, New Taipei City, Taiwan

^15^Department of Chemical Engineering, Stanford University, Stanford, CA 94305

^16^Department of Cell and Molecular Biology, College of Medicine, Chang Gung University, Taoyuan, Taiwan

^17^Graduate Institute of Biomedical Sciences, College of Medicine, Chang Gung University, Taoyuan, Taiwan

^18^Division of Hematology-Oncology, Department of Internal Medicine, Chang Gung Memorial Hospital at Linkou, Taoyuan, Taiwan

^19^Department of Biochemistry, School of Medicine, Kaohsiung Medical University, Kaohsiung, Taiwan

^20^Chong Hin Loon Memorial Cancer and Biotherapy Research Center, National Yang Ming Chiao Tung University, Taipei, Taiwan

**#These authors contributed equally to this study**.

*Corresponding authors

Yuh-Min Chen: email: ymchen@vghtpe.gov.tw ORCID: 0000-0003-0585-8463

Hsuan Liu: email: liu-hsuan@mail.cgu.edu.tw ORCID: 0000-0003-4691-2518

Chi-Ying F. Huang: email: cyhuang5@nycu.edu.tw ORCID: 0000-0003-4898-4937

