# Supplementary material for: Utilizing TP53 hotspot mutations as effective predictors of gemcitabine treatment outcome in non-small-cell lung cancer
Source: Cell Death Discov. 2025 Jan 27;11:26. doi: 10.1038/s41420-025-02300-7 (PMC11772833; doi:10.1038/s41420-025-02300-7)
Supplement: Supplementary file 1 — sup figure [file 41420_2025_2300_MOESM1_ESM.pdf]

Supplement figures

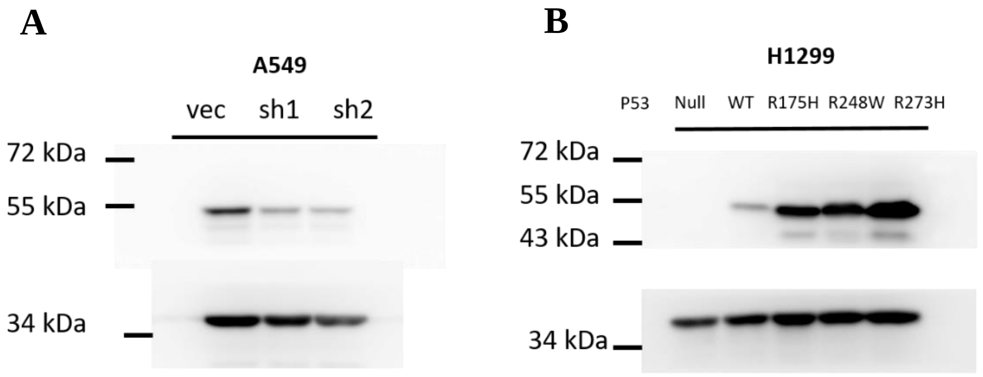

Supplement figure 1: (**A**) Knockdown *TP53* efficacy in A549 cells was evaluated by western blot. (B) Overexpression of *TP53* either mutations or wild type efficacy in H1299 cells was evaluated by western blot.

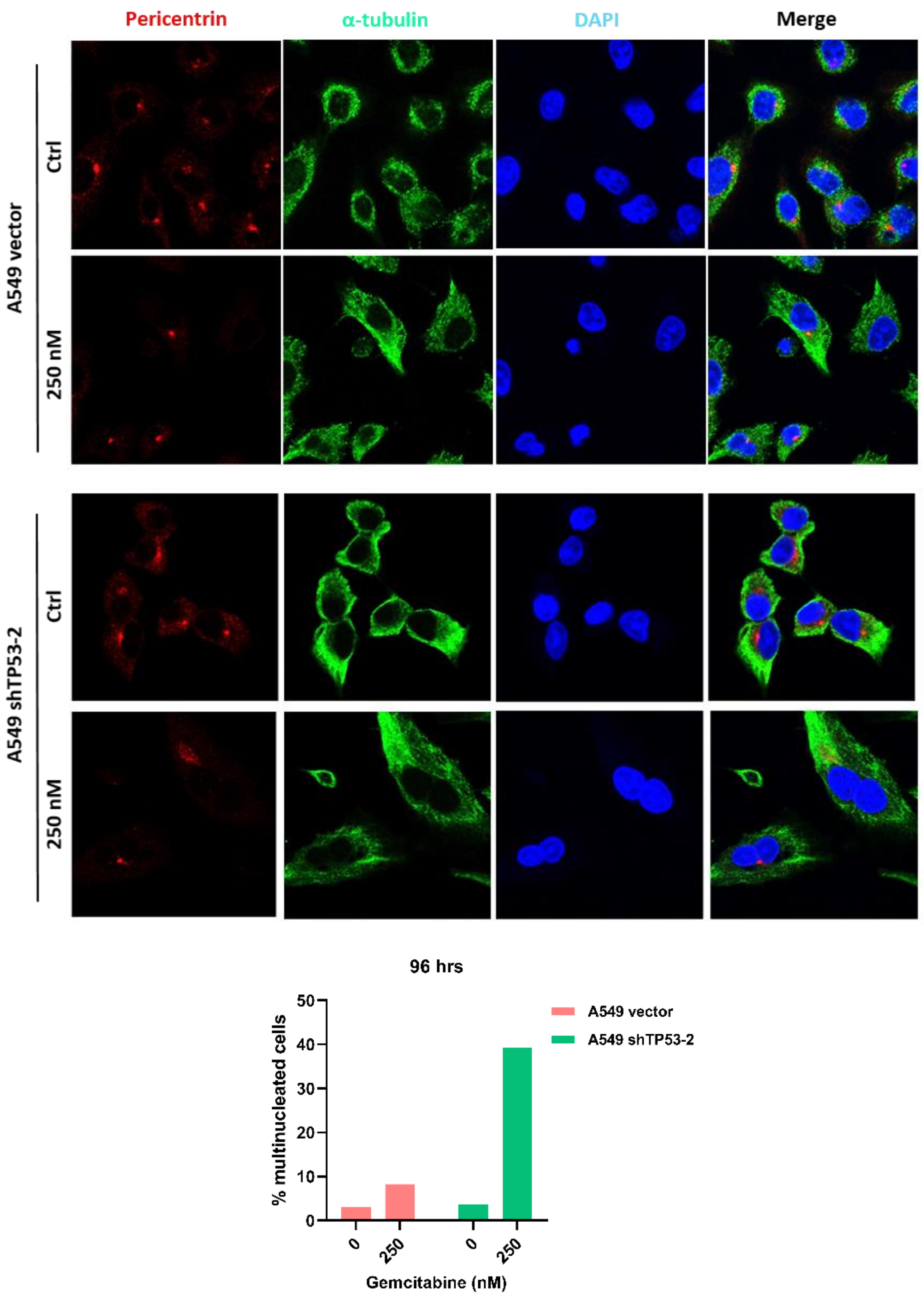

Supplement figure 2: Gemcitabine-Induced Formation of Multinucleated Cells in A549 *TP53* Knockdown Cell Line. This figure illustrates the effects of gemcitabine treatment on A549 *TP53* knockdown cells, leading to the formation of multinucleated cells. Representative images show the presence of abnormal centrosomes and

multinucleated cells. Pericentrin, serving as a centrosome marker, is depicted in red; α-tubulin, a protein associated with microtubules, is shown in green; and DAPI, used to stain chromatin, appears in blue. After 96 hours of gemcitabine treatment, an increase in the number of multinucleated cells is observed in A549 *TP53* knockdown cells. The data are visually depicted to highlight the effects of gemcitabine on cell morphology and centrosome dynamics in A549 *TP53* knockdown cell.

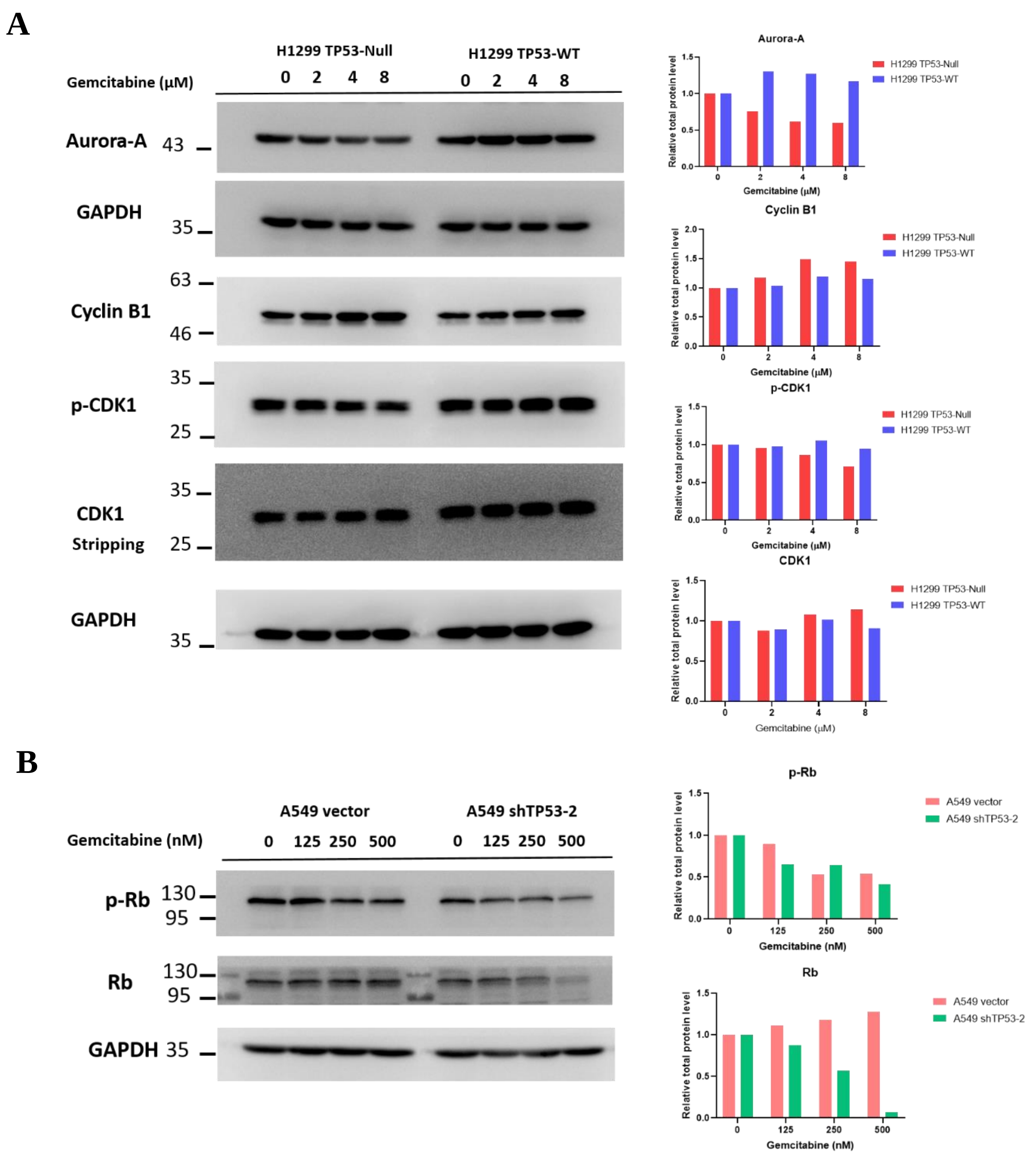

Supplement figure 3: Protein markers involved in cell cycle arrest at G2/M phase and mitosis processes. (A) Western blot analysis of H1299 cell lines (TP53-Null and TP53-WT) in response to gemcitabine treatment after 48 hours. (B) Western blot analysis of A549 cell lines (vector and shTP53-2) in response to gemcitabine treatment after 48 hours.

A

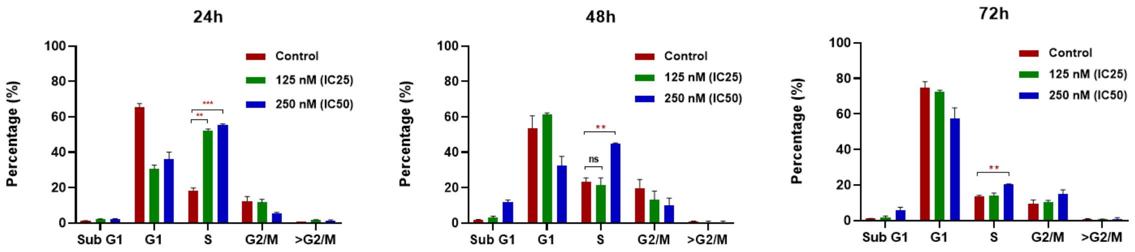

B

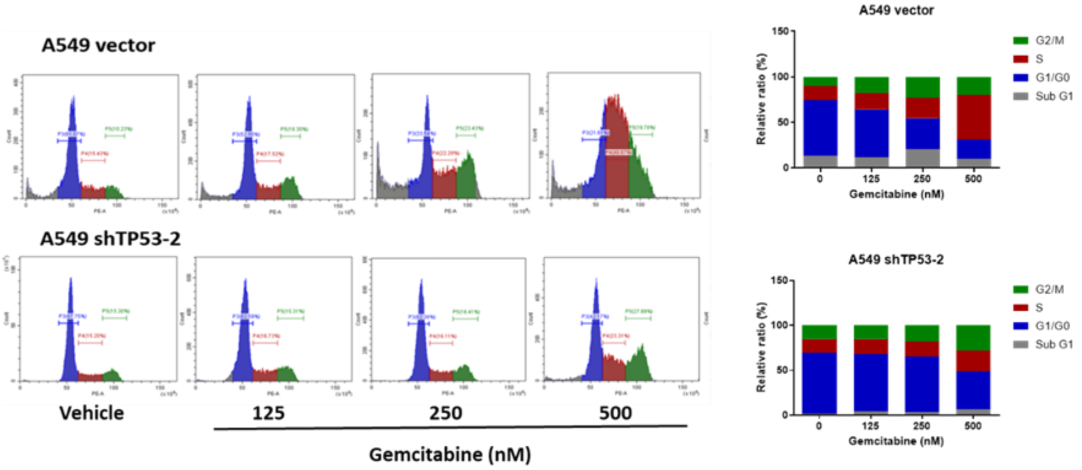

Supplement figure 4: Treatment with gemcitabine induces cell cycle arrest at the S phase in *TP53*-wild type A549 cells, followed by a reversal of the arrest. (A) A549 *TP53*-WT cells treated with gemcitabine were arrested in the S phase after 24 hours and subsequently reversed the cell cycle arrest at 48 and 72 hours in a dose-dependent manner. (B) A comparison between A549 *TP53*-WT (vector) cells and *TP53* knockdown (shTP53-2) cells at different doses of gemcitabine showed that only vector cells were arrested at the S phase.

Fig 6D

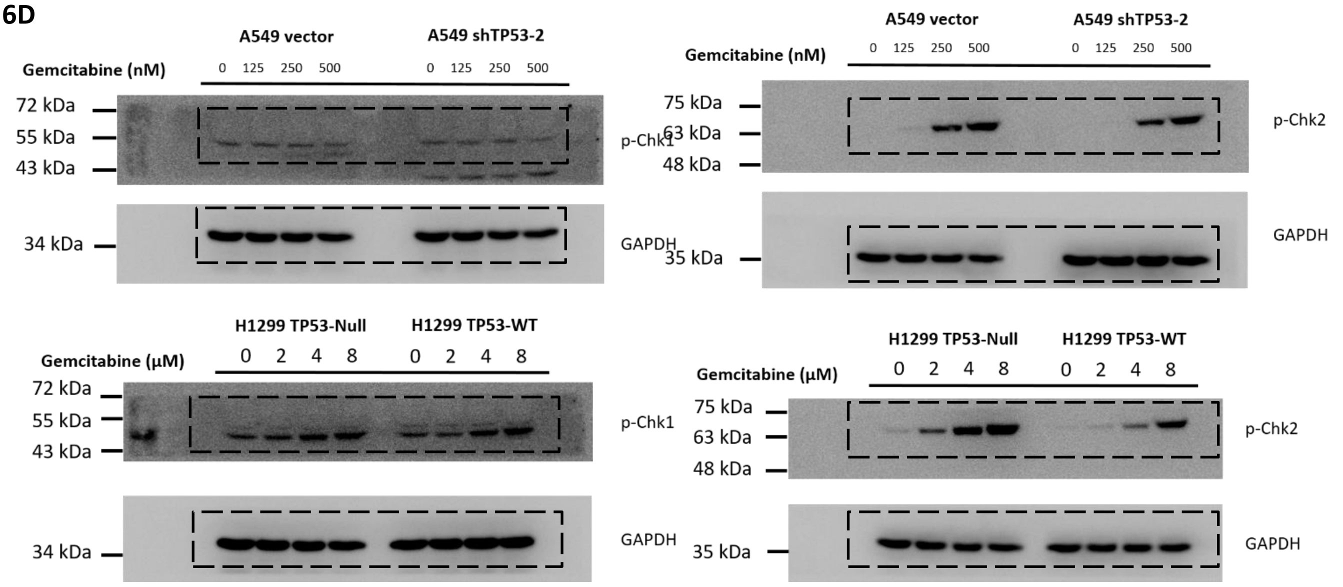
A549 vector
A549 shTP53-2
Gemcitabine (nM)
0 125 250 500 0 125 250 500
72 kDa
55 kDa
43 kDa
p-Chk1
34 kDa
GAPDH
75 kDa
63 kDa
48 kDa
p-Chk2
35 kDa
H1299 TP53-Null
H1299 TP53-WT
Gemcitabine (μM)
0 2 4 8 0 2 4 8

Fig 6E

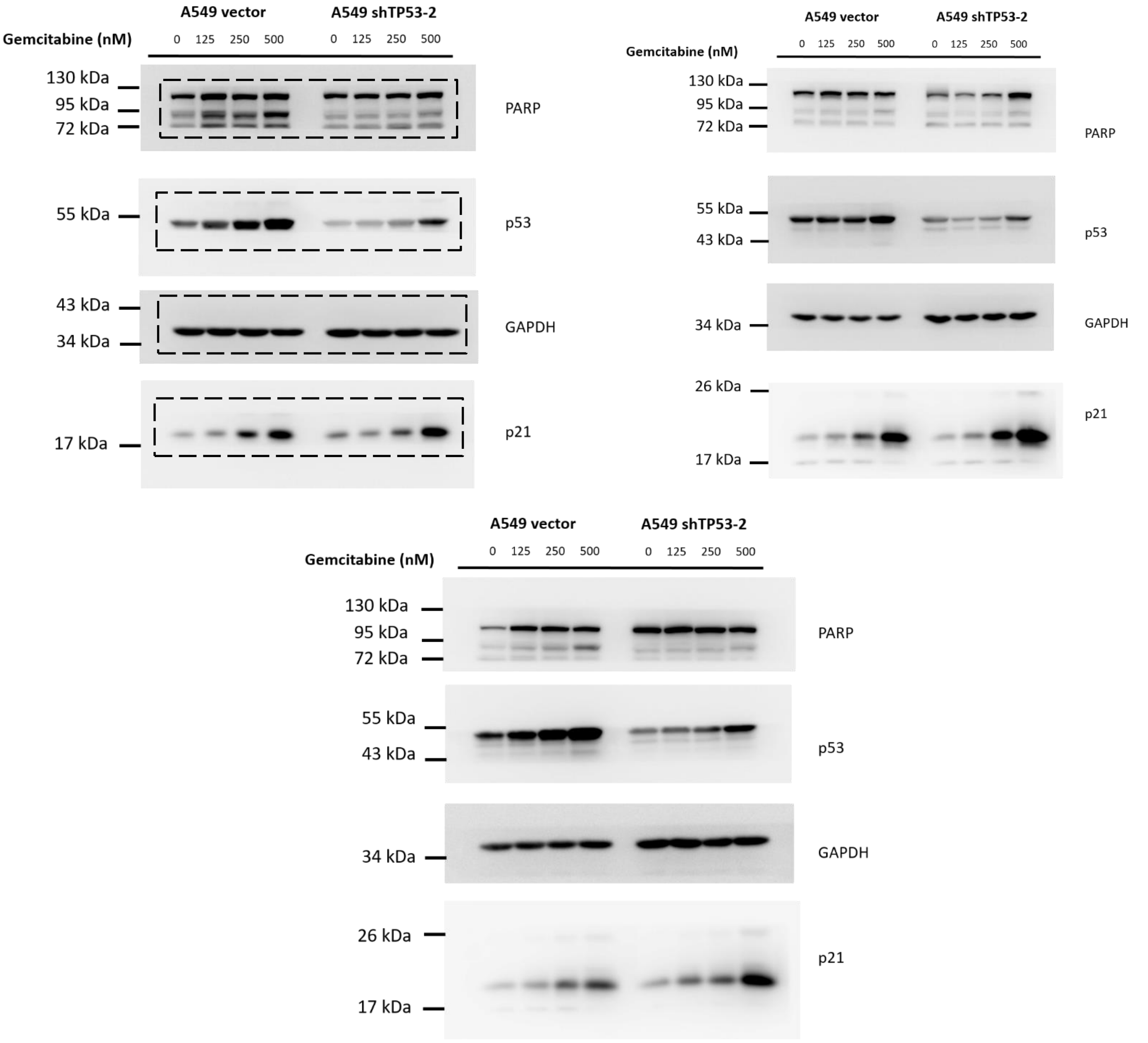
A549 vector
A549 shTP53-2
Gemcitabine (nM)
0 125 250 500 0 125 250 500
130 kDa
95 kDa
72 kDa
PARP
55 kDa
43 kDa
p53
34 kDa
GAPDH
26 kDa
17 kDa
p21

Fig 6F

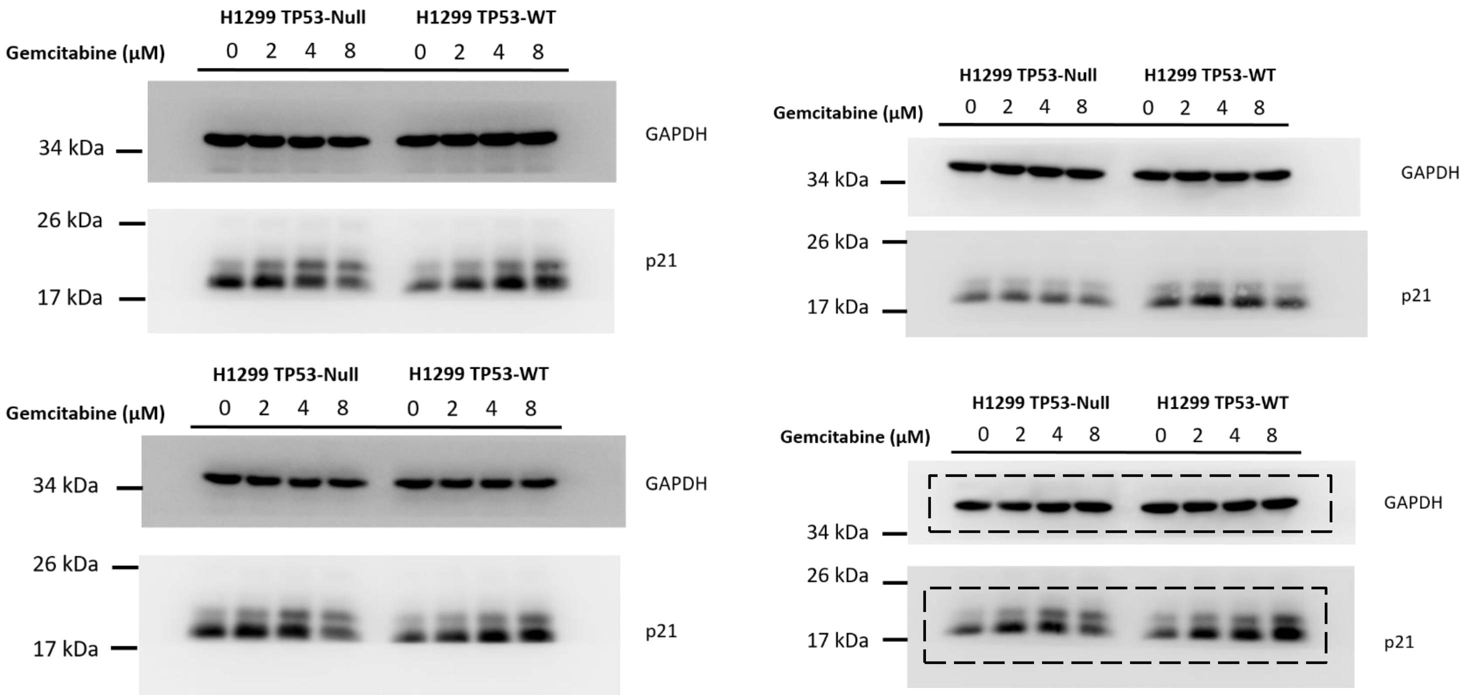
H1299 TP53-Null
H1299 TP53-WT
Gemcitabine (μM)
0 2 4 8 0 2 4 8
34 kDa
GAPDH
26 kDa
17 kDa
p21
H1299 TP53-Null
H1299 TP53-WT
Gemcitabine (μM)
0 2 4 8 0 2 4 8
34 kDa
GAPDH
26 kDa
17 kDa
p21
H1299 TP53-Null
H1299 TP53-WT
Gemcitabine (μM)
0 2 4 8 0 2 4 8
34 kDa
GAPDH
26 kDa
17 kDa
p21
H1299 TP53-Null
H1299 TP53-WT
Gemcitabine (μM)
0 2 4 8 0 2 4 8
GAPDH
34 kDa
26 kDa
17 kDa
p21

Fig 5D

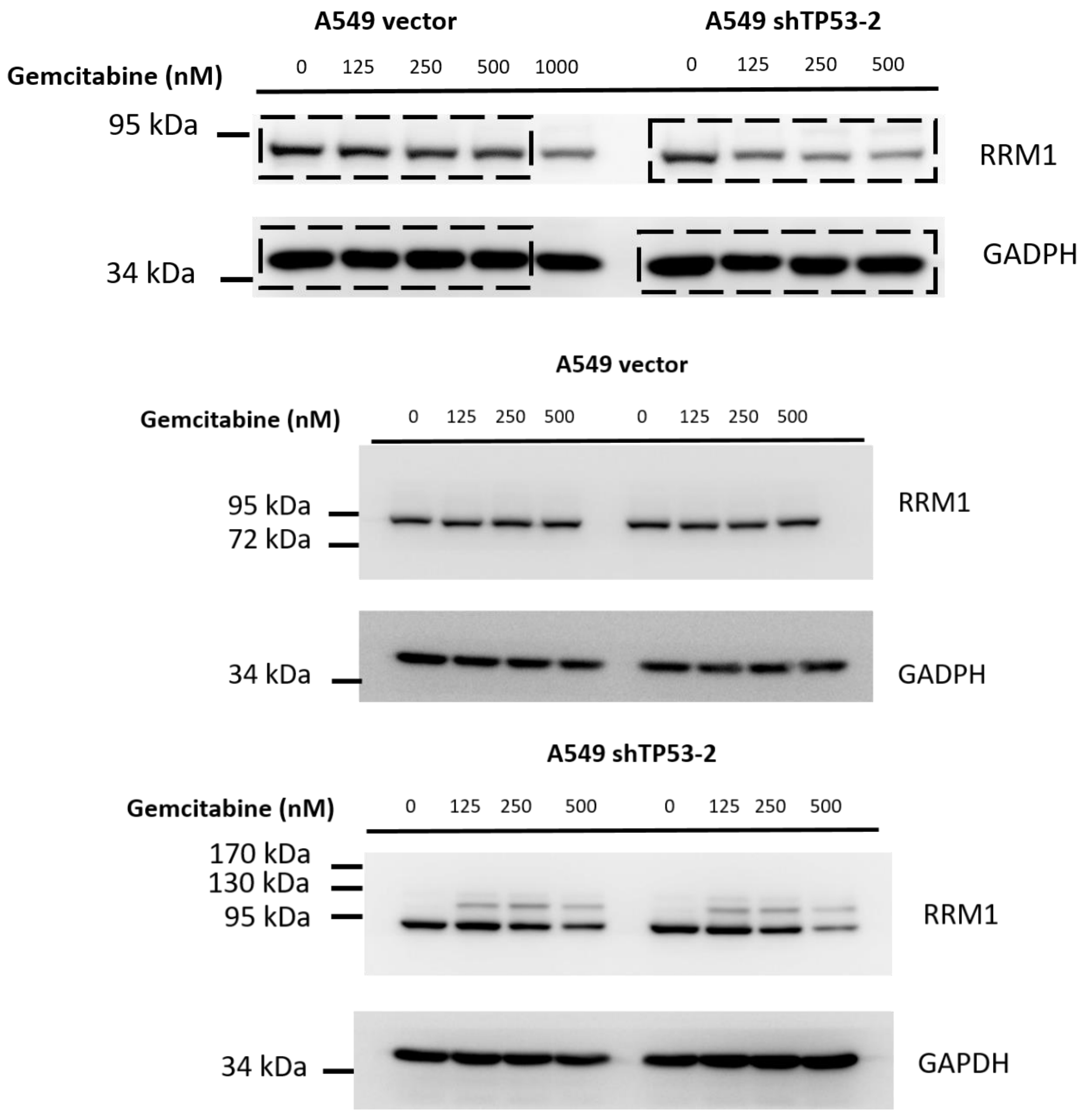
A549 vector
A549 shTP53-2
Gemcitabine (nM)
0 125 250 500 1000
0 125 250 500
95 kDa
RRM1
34 kDa
GADPH
A549 vector
Gemcitabine (nM)
0 125 250 500 0 125 250 500
95 kDa
72 kDa
RRM1
34 kDa
GADPH
A549 shTP53-2
Gemcitabine (nM)
0 125 250 500 0 125 250 500
170 kDa
130 kDa
95 kDa
RRM1
34 kDa
GAPDH

Supplement figure 5: Western blot replicates as cropped and uncropped images. Uncropped full-length pictures of western blotting membranes presented in the main Fig. 6 D, E, F, and Fig. 5D . Membranes were often cut to enable blotting for multiple antibodies.
