# Supplementary figures and images for: Utilizing TP53 hotspot mutations as effective predictors of gemcitabine treatment outcome in non-small-cell lung cancer
Source: Cell Death Discov. 2025 Jan 27;11:26. doi: 10.1038/s41420-025-02300-7 (PMC11772833; doi:10.1038/s41420-025-02300-7)

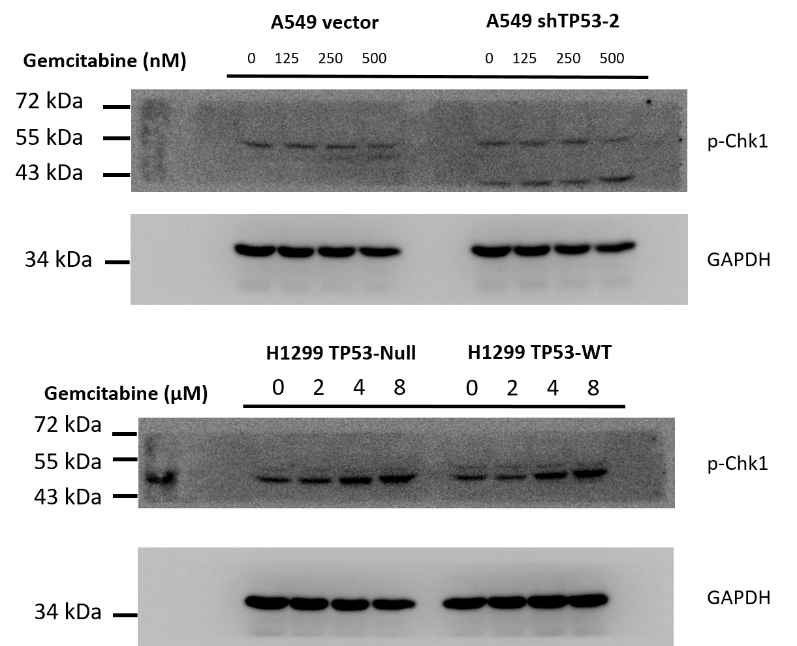

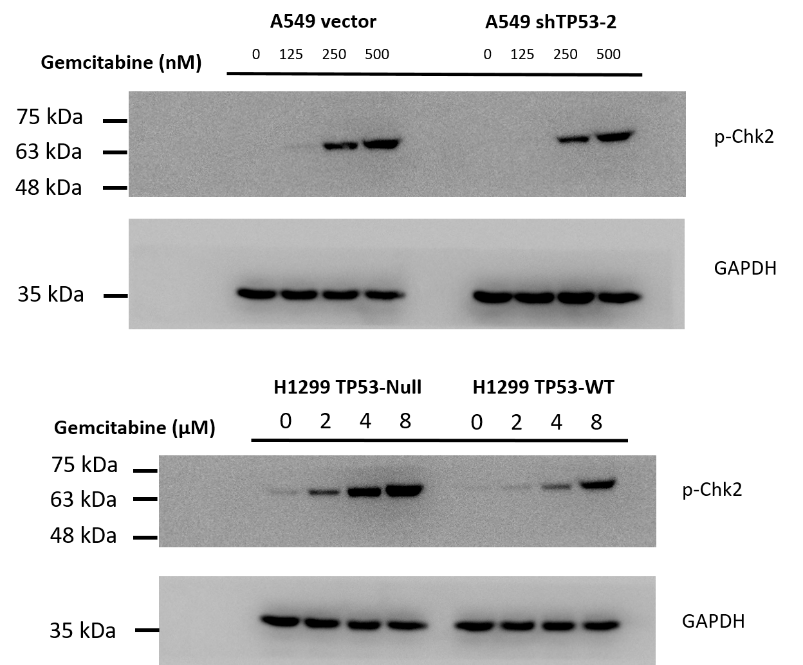

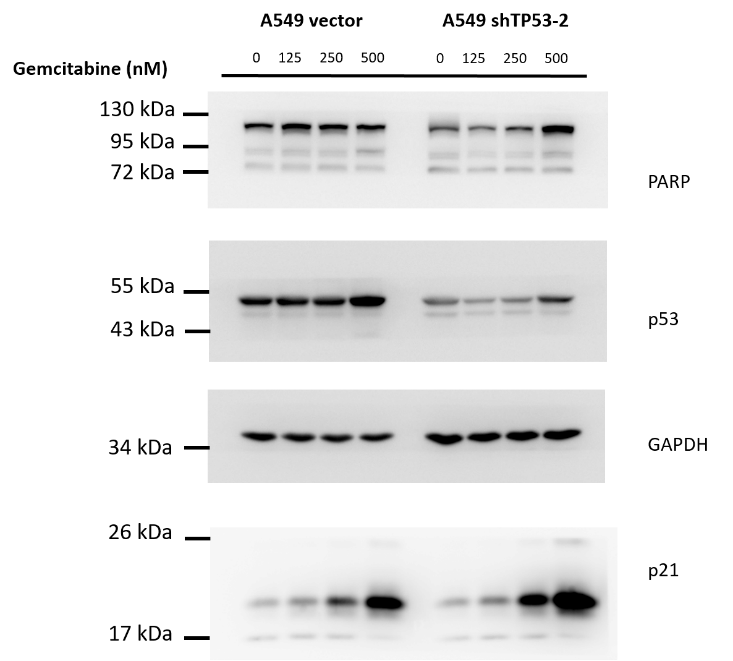


**Fig 6E**

**Fig 6D**


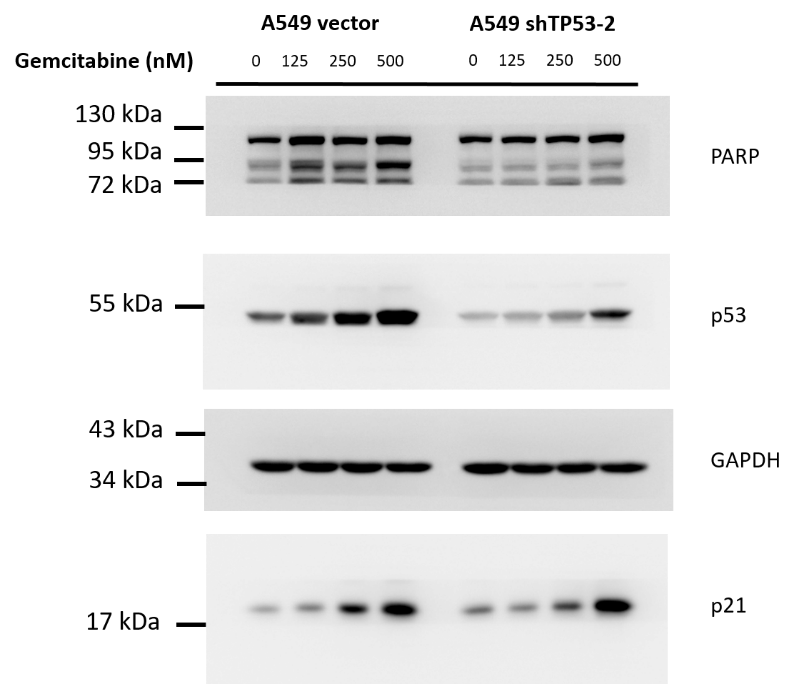


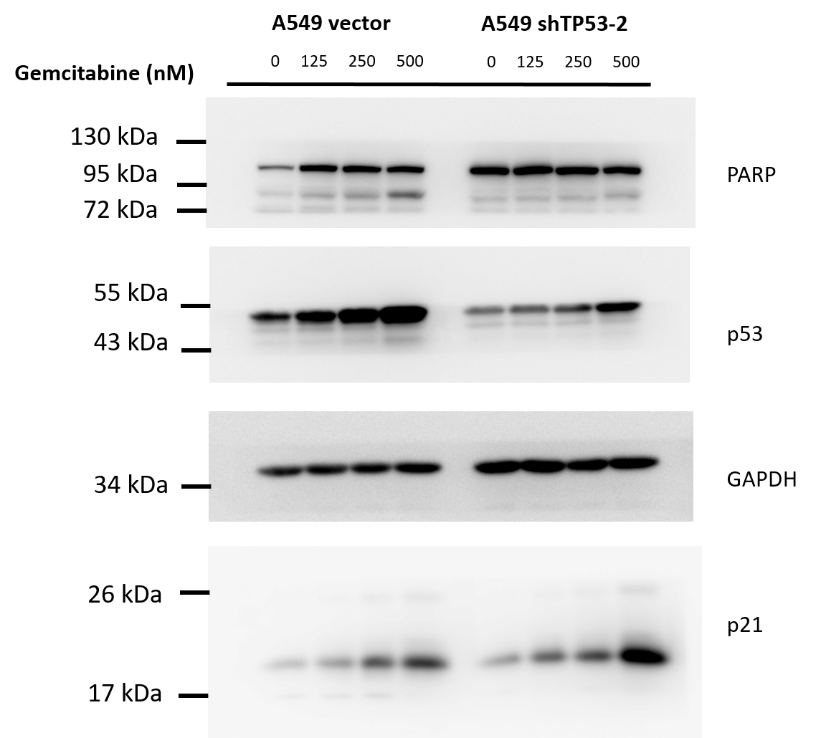


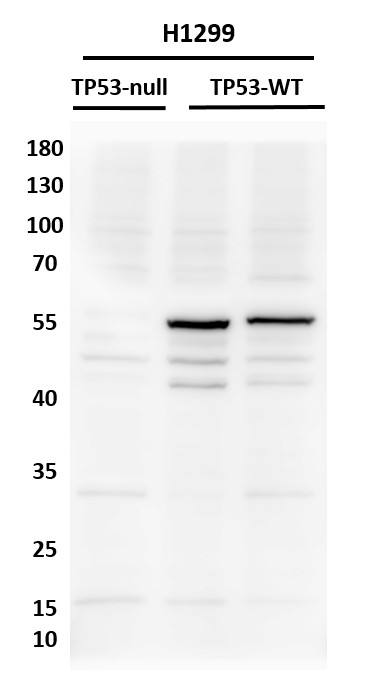

Supplement: Supplementary file 3 — original data [file 41420_2025_2300_MOESM3_ESM.docx]
